# Novel Human Embryonic Stem Cell Regulators Identified by Conserved and Distinct CpG Island Methylation State

**DOI:** 10.1371/journal.pone.0131102

**Published:** 2015-07-07

**Authors:** Steve Pells, Eirini Koutsouraki, Sofia Morfopoulou, Sara Valencia-Cadavid, Simon R. Tomlinson, Ravi Kalathur, Matthias E. Futschik, Paul A. De Sousa

**Affiliations:** 1 MRC Centre for Regenerative Medicine, School of Clinical Studies, University of Edinburgh, Edinburgh, EH16 4SB, United Kingdom; 2 Centre for Clinical Brain Sciences, University of Edinburgh, Edinburgh, EH16 4SB, United Kingdom; 3 Centre for Molecular and Structural Biomedicine, University of Algarve, 8005–139, Faro, Portugal; Michigan State University, UNITED STATES

## Abstract

Human embryonic stem cells (hESCs) undergo epigenetic changes *in vitro* which may compromise function, so an epigenetic pluripotency “signature” would be invaluable for line validation. We assessed Cytosine-phosphate-Guanine Island (CGI) methylation in hESCs by genomic DNA hybridisation to a CGI array, and saw substantial variation in CGI methylation between lines. Comparison of hESC CGI methylation profiles to corresponding somatic tissue data and hESC mRNA expression profiles identified a conserved hESC-specific methylation pattern associated with expressed genes. Transcriptional repressors and activators were over-represented amongst genes whose associated CGIs were methylated or unmethylated specifically in hESCs, respectively. Knockdown of candidate transcriptional regulators (HMGA1, GLIS2, PFDN5) induced differentiation in hESCs, whereas ectopic expression in fibroblasts modulated iPSC colony formation. Chromatin immunoprecipitation confirmed interaction between the candidates and the core pluripotency transcription factor network. We thus identify novel pluripotency genes on the basis of a conserved and distinct epigenetic configuration in human stem cells.

## Introduction

The application of human embryonic stem cells (hESCs) to regenerative medicine relies on maintaining appropriate gene expression controlling self renewal or lineage specification *in vitro*. Epigenetic modifications of DNA and chromatin control the expression patterns that define cellular identity and function during development and in differentiated tissues [1]. Methylation of cytosine in cytosine-phosphate-guanine (CpG) dinucleotides is an epigenetic mark conferring stability on gene expression states, notably by the establishment of a silent chromatin state [2]. In normal sequence, where CpGs are relatively infrequent (~ 1 per 100 bp), most CpGs are methylated, but, in Cytosine-phosphate-Guanine Islands (CGIs), where CpGs typically comprise about 1 in 10 bp for sequences ~1 to 2 kb in length, they are usually unmethylated. About 70% of CGIs are associated with gene promoters [3]. While CGIs are normally unmethylated, exceptions include those associated with imprinted genes [4], genes subject to X-chromosome inactivation [5], and transposable elements [6, 7].

Genetic changes are common in hESCs as a cell line adapts to culture [8, 9], and hESCs are also neither epigenetically homogenous nor stable *in vitro*. Gene promoter methylation and X-inactivation states vary between cell lines and even between different cultures of the same line [10] [11–17]. The same is true for cells induced into pluripotency, which can retain a residual epigenetic memory of their origin [18–22]. As not all epigenetic variations affect cell behaviour, it is important to identify those relevant to a pluripotent phenotype.

DNA methylation in hESCs has been studied by various methods [12, 23] [24] [25]. We have used a human CGI array (> 17,000 CGIs) to identify CGIs that are either methylated or unmethylated in hESCs, and identify CGI methylation patterns conserved between hESC lines and distinct from differentiated tissues assessed previously [26]. These conserved patterns define putative biomarkers of the pluripotent state at an epigenetic level; that is, CGIs whose methylation status is apparently unique to hESCs. Functional roles for selected candidates in regulation of stem cell phenotype were confirmed by small RNA-mediated interference and modulation of somatic cell reprogramming frequency. We propose a model whereby transcriptional communication between “secondary” pluripotency-associated factors such as the epigenetically-defined biomarkers described here and the core pluripotency network ensures that expression of both groups of factors is achieved in pluripotent cells and modulated precisely in differentiating cells.

## Materials and Methods

### Human embryonic stem cell lines

HESC lines were derived under license from the UK Human Fertilisation and Embryology Authority (R0136). Their identity, provenance and culture conditions are summarised in table A in S1 File and detailed previously [27, 28].

### CGI analysis

For each hESC line, gDNA was prepared from 2 biological replicates (RCM1) or 4 biological replicates (RH1, 3 and 4) of 10^5^ cells per replicate. gDNA was purified, digested and ligated to "catch linkers" prior to MBD2 column binding, elution and array hybridisation, all as described previously [26].

### mRNA expression analysis

Expression analysis was performed on samples concurrent with those used to evaluate the CGI methylation of gDNA. RNA was prepared and array data analysed as described [27].

Annotation from Gene Ontology was processed through the Bioconductor package GO.db. Statistical significance for overrepresentation of expressed sets of genes within GO categories were derived through the hypergeometric test (equivalent to Fisher´s exact test). P-values were adjusted for multiple testing with the Benjamini-Hochberg procedure.

### Screening of candidate gene function by RNA interference

Genes were knocked down in hESCs by transfection of siRNA molecules with RNAiMAX Lipofectamine (Invitrogen) (S8 Fig). SiRNA sequences are shown in Table M in S1 File.

### RT-qPCR Analysis

Gene expression analyses were performed as described [29]. Table O in S1 File gives primer sequences.

### Immunocytochemistry

For OCT4 and NANOG immunohistochemistry, hESCs were fixed and immunostained as described [30]. Immunostaining for methylated and hydroxymethylated DNA used a protocol adapted from that previously reported [31] to permit costaining with DAPI and mC or 5-hmC visualisation.

### 5-hmC ELISA

The 5-hmC content of genomic DNA was measured by a hydroxymethylated DNA quantification kit (Quest 5-hmC DNA ELISA Kit, ZYMO Research) according to manufacturer’s protocol.

### Chromatin Immunoprecipitation

HESC chromatin was prepared using the ChIP-IT High Sensitivity kit (Active Motif), with approximately 50,000 cell equivalents and 4 μg antibody to OCT4 (Santa Cruz SC-8628) was used per reaction. Controls and data from the ChIP-IT qPCR analysis kit (Active Motif) were used to create a standard curve and calculate the number of binding events per 1000 cells in each sample. Primer sequences for the promoter regions of HMGA1, GLIS2 and PFDN5 incorporating OCT4 binding sites are shown in Table P in S1 File.

### Reprogramming of Human Dermal Fibroblasts

10 independent transfections were carried out for each condition to ensure a statistically powerful experiment. Briefly, 2x10^5^ HDFs (≤ passage 8; Cascade Biologics C0045C) were harvested by trypsinisation and transfected in a 20 μl reaction containing 0.04 pmol of each plasmid (pCXLE-OCT4sh53, pCXLE-UL, pCXLE-SK [32] and pCXLE-GW-HMGA1, pCXLE-GW-GLIS2 and/or pCXLE-GW-PFDN5 (S13 Fig) using a Nucleofector X Unit running program EN150 and nucleofection solution P2 (Lonza). Cells were plated out and cultured in fibroblast medium (KO-DMEM, 10% FCS, L-gln, penicillin & streptomycin) for 5–8 days, then replated on Matrigel and cultured in mTeSR1 medium until colonies appeared. Plates were fixed and stained for alkaline phosphatase activity using the Stemgent AP2 kit according to the manufacturer’s instructions.

### Statistical Analyses

Statistical analyses were performed in Graphpad Prism, using unpaired t-tests or ANOVA, as appropriate.

## Results

### CGI Array Hybridisation of hESC MAP-gDNA

As a pilot study of the significance of CGI methylation to pluripotency, 4 hESC lines, differing in provenance, sex, passage number and culture conditions were assessed to identify a conserved pattern of CGI methylation status (Table A in S1 File; summary of hESC lines employed in this study). Three of these lines (RH1, RH3 and RCM1) were female, and one (RH4) was male. To investigate hESC line CGI methylation status, we probed an array of biologically-defined CGIs used previously to study tissue-specific methylation[26] (S3 Table). A CGI was designated as gene-associated (GA-CGI) if it mapped to within 1.5 kb of an annotated gene or overlapped with the gene itself. Thus of 17,387 CGIs on the array, 13,657 were gene-associated (78.5%; S3 Table).

Control (unpurified) input gDNA comparisons between lines gave similar levels of CGI fragment: probe hybridisation for the autosomes (within 0.5 log_2_), as expected for euploid human cell lines (S2 Fig). When RH4 (male) was compared with female lines, the X chromosome showed significantly weaker hybridisation in RH4 whereas the Y chromosome showed stronger hybridisation (S2 Fig). This difference in DNA hybridisation signal is expected for the sex chromosomes from lines of different sexes, and confirmed the CGI array system resolution to within 2-fold. To identify methylated CGIs, arrays were probed with MBD2 methyl-binding domain column-purified genomic DNA (MAP-gDNA), with hybridisation signal compared against total gDNA (See S1 Fig for schematic overview). A CGI was designated as methylated (Me-CGI) when the M value (log_2_[*MAP-gDNA/Total gDNA*]) was ≥ 1.5 and the adjusted p value ≤ 0.1; otherwise it was designated as unmethylated. The complete CGI methylation dataset is available (S5 Table). Examples of CGI methylation or lack thereof in different cell lines identified by the CGI array were confirmed by sequencing of fragments amplified from bisulphite-treated gDNA (S3 Fig).

### CGI Methylation of Human Embryonic Stem Cells

**Fig 1:**
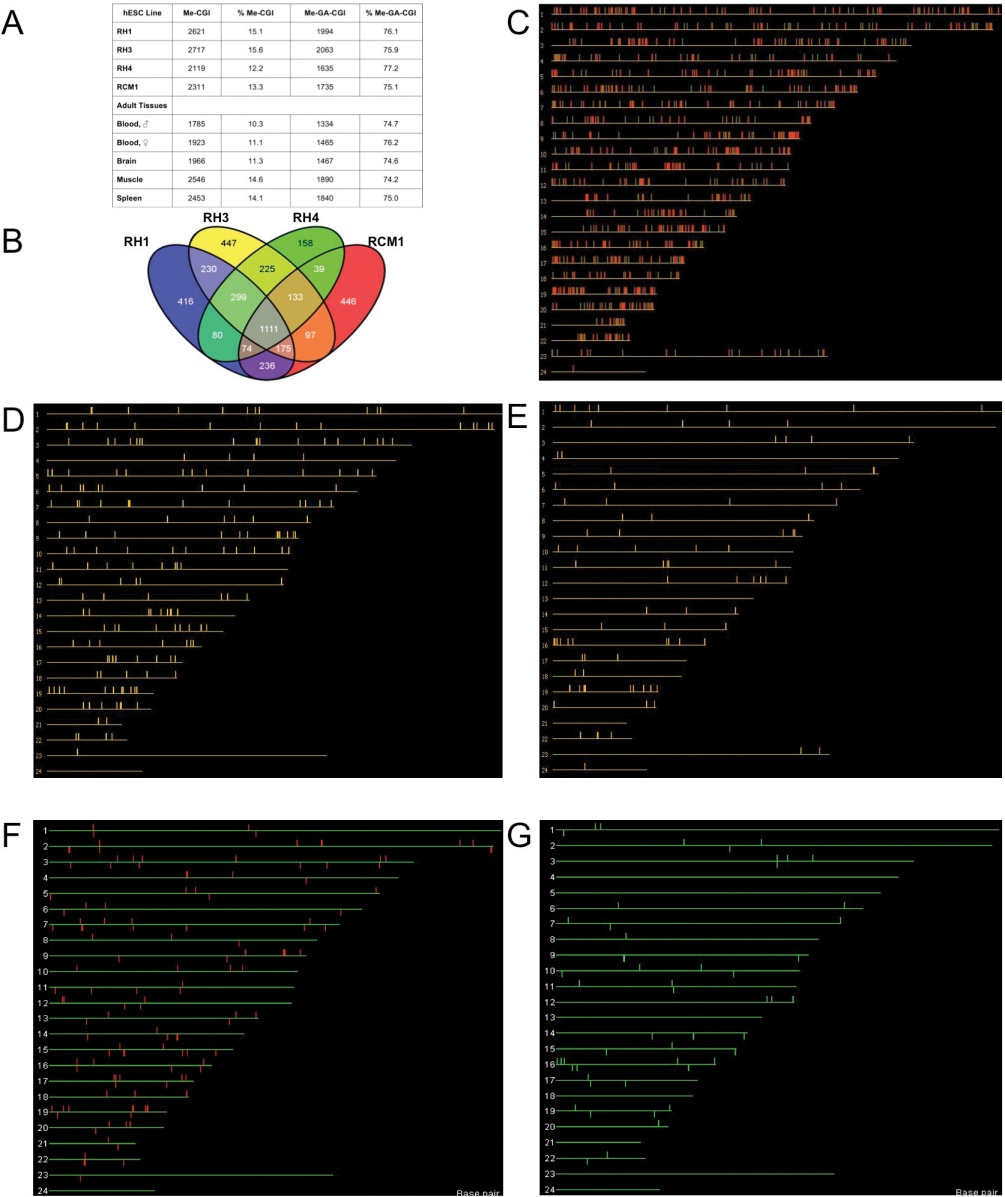
Genome-wide CGI methylation analysis of hESC lines. (A) Table showing the number of methylated CGIs (Me-CGI) in each hESC line, and for adult somatic tissues (Illingworth et al., 2008), the percentage of methylated CGIs (% Me-CGI), number of methylated gene-associated CGIs (Me-GA-CGI) and the percentage of methylated CGIs that are gene-associated (% Me-GA-CGI). Me-CGIs are given in the supplementary file S1 Table. (B) Venn Diagram shows heterogeneity of hESC CGI methylation. 1111 CGIs are methylated in all 4 lines. (C-G) Genome maps depicting locations of various CGI groups: (C) Me-CGIs in hESC lines (red, gene-associated; green, not gene-associated). (D) 201 GA-CGIs methylated in hESCs but unmethylated in somatic tissues. (E) 98 GA-CGIs unmethylated in hESCs but methylated in somatic tissues. (F) hESC-expressed genes whose associated CGIs are hESC-methylated and unmethylated in somatic tissues. (G) hESC-expressed genes whose associated CGIs are hESC-unmethylated and methylated in somatic tissues. Autosomes ordered 1–22, 23 = X, 24 = Y.

HESC MAP-gDNA hybridisation data showed that 12–16% of CGIs were methylated in hESCs, depending on cell line (2119 Me-CGIs in RH4 to 2717 in RH3). For consistency, somatic tissue data reported previously[26] were reanalysed in parallel. Similar proportions of CGIs were methylated in somatic tissues as in hESCs, varying from 10–14% (1785 Me-CGIs in male blood to 2546 Me-CGIs in muscle [Fig 1A; corresponding numbers and proportions of unmethylated CGIs are listed in table Q in S1 File]). Overall CGI methylation levels are thus similar in hESCs and somatic tissues. There was no significant difference in CGI methylation rates between hESC lines and somatic tissues for CGIs generally, or for gene-associated CGIs specifically (P = 0.142 in both cases; Kruskall-Wallis).

CGI methylation varied between hESC lines. Comparison of Me-CGIs between the female lines RH1, RH3 and RCM1 revealed heterogeneity between pairs of lines (S4 Fig). There were frequent differences greater than 0.5-fold throughout the genome between RH1 and RCM1, and between RH3 and RCM1 (S4A and S4B Fig). Individual CGIs were either hypermethylated (red, M>1.5; difference between lines >0.75) or hypomethylated (blue, M<1.5; difference between lines >0.75) in RH1 and RH3 cf. RCM1, rather than one cell line in a comparison being consistently hypo- or hypermethylated with respect to the other. However, the sibling lines RH1 and RH3 showed similar levels of autosomal CGI methylation (S4C Fig). In the case of the X chromosome, RH1 CGI methylation was generally higher than that of RH3 (S4C and S4D Fig), corresponding to methylation of 137 X-linked GA-CGIs in RH1, compared to 39 for RH3, similar to that observed for the male line RH4 (33 CGIs). The female line RCM1 had 138 X-linked Me-GA-CGIs, similar to RH1 (Table B in S1 File; methylation of X-linked gene-associated CGIs in hESC lines). RH3 and RH4 (male) have similar levels of X chromosomal and autosomal CGI methylation (Table B in S1 File, no significant difference in X-linked Me-CGIs from expected for RH3 or RH4). X-linked CGI methylation for RH1 and RCM1 was significantly higher than expected (P<<0.001 for both lines; Χ^2^ = 203.3 [RH1] or 265.8 [RCM1]).

Despite the heterogeneity of hESC CGI methylation, correlation of Me-CGI lists for different lines identified 1111 CGIs (40.8%–52.4% Me-CGIs, depending on cell line) methylated in all hESC lines tested (Fig 1B and 1C and S1 Table and S2 Table) 311 of these CGIs are also methylated in somatic tissues. The set of 1111 CGIs was reduced to 1079 by removal of 32 CGIs for which there was more than one reporter which behaved differently in different lines (Table C in S1 File). 828 CGIs methylated in all hESC lines are gene-associated, significantly fewer than the expected proportion (78.5%) of methylated gene-associated CGIs (Me-GA-CGIs, 873 expected, Χ^2^ = 10.4, P = 0.0012). These 828 Me-GA-CGIs are associated with 891 genes (S2 Table). No localisation of CGI methylation to particular chromosomes was apparent (Fig 1C and 1D, P = 0.371, Χ^2^ = 24.6). Comparison of CGI sets that were uniformly methylated or unmethylated in hESCs with somatic tissues identified two subsets whose methylation patterns were specific to hESCs. 201 GA-CGIs (associated with 220 genes, reduced to 216 as 4 genes were associated with multiple, differentially methylated, CGIs) were methylated in hESCs and unmethylated in adult tissues; conversely, 98 CGIs (associated with 113 genes) were unmethylated in hESCs and methylated in somatic tissues (Fig 1D and 1E and Table D in S1 File). As with the Me-CGIs, genes with a CGI methylated in hESCs and unmethylated in somatic tissues showed no significant chromosomal localisation (hESC-Me-GA-CGIs, Χ^2^ = 22.6, P = 0.244), nor did genes with an hESC-unmethylated/somatic methylated CGI (hESC-UnMe-GA-CGIs, Χ^2^ = 25.199, P = 0.288).

### Transcriptome Analysis of hESCs

RNA transcriptome data were obtained for the three female hESC lines RH1, RH3 and RCM1. RH4 data were not included as RH4 is male and thus hemizygous for X, unaffected by X chromosome inactivation but expressing Y-linked genes.

Triplicate RNA samples from independent biological replicates concurrent with those used to prepare gDNA for CGI methylation analysis were hybridised to Affymetrix U133Plus2 GeneChips. Similar expression distributions were seen for all arrays. Arrays clustered to individual cell lines (S5 Fig), indicating high reproducibility of microarray data. Whilst overall expression patterns were similar for the three lines (Pearson correlation coefficient r>0.99), X-linked expression for RH3 was consistently higher than RH1 and RCM1, which were similar to each other (S6 Fig). The complete transcriptome dataset is available as supplementary S6 Table.

### Correlation of CGI Status with Expression

Correlation of expression data with CGI methylation data defined two smaller gene sets. Of 216 genes associated with 201 hESC-Me-GA-CGIs (1.47% of GA-CGIs), 128 (59.3%) were expressed in all three lines (Fig 1F, and Table E and Table F in S1 File). Similarly, of 109 genes associated with 98 hESC-UnMe-GA-CGIs (0.72% GA-CGIs), 56 (57.1%) were expressed in all three lines (Fig 1G, and Table E and Table G in S1 File). As with CGI methylation generally, no localisation of expressed genes with a methylated CGI was apparent (Fig 1F; expressed hESC-Me-GA-CGIs, Χ^2^ = 25.694, P = 0.265). However, distribution of expressed genes with an associated hESC-unmethylated CGI was apparently non-random (Fig 1G; expressed hESC-UnMe-GA-CGIs, Χ^2^ = 33.886, P = 0.05), due to enrichment of chromosome 16 genes (9 observed; 2.5 expected, Χ^2^ = 18.298, P = 1.9x10^-5^).

There was a significant correlation in only three cases of differential methylation and differential expression between a pair of hESC lines (RH1, RH3, RCM1; Table H in S1 File, differential CGI methylation and gene expression in female hESC lines). These were genes whose associated CGI was methylated in RH1 but unmethylated in RH3 and that were significantly more highly-expressed in RH3, genes whose associated CGI was methylated in RCM1, unmethylated in RH1 and that were more highly expressed in RH1, and genes whose associated CGI was methylated in RCM1, unmethylated in RH3 and that were more highly expressed in RH3. Thus the methylation and expression states were consistent with the established association of DNA methylation with gene silencing. Of 80 genes whose associated CGI was methylated in RH1 but unmethylated in RH3 that were expressed in RH3 but not RH1, 50 were X-linked, a very significant result (Table I in S1 File, association of X chromosome with differential methylation and expression in female hESC lines). Similarly, X-linked GA-CGIs differentially methylated between female lines and more highly expressed in the unmethylated partner were significantly overrepresented in the RCM1: RH1 and RCM1: RH3 comparisons. (Table I in S1 File and S6 Fig).

### Gene Ontology Identifies Candidate Epigenetic Biomarkers of hESCs

**Fig 2:**
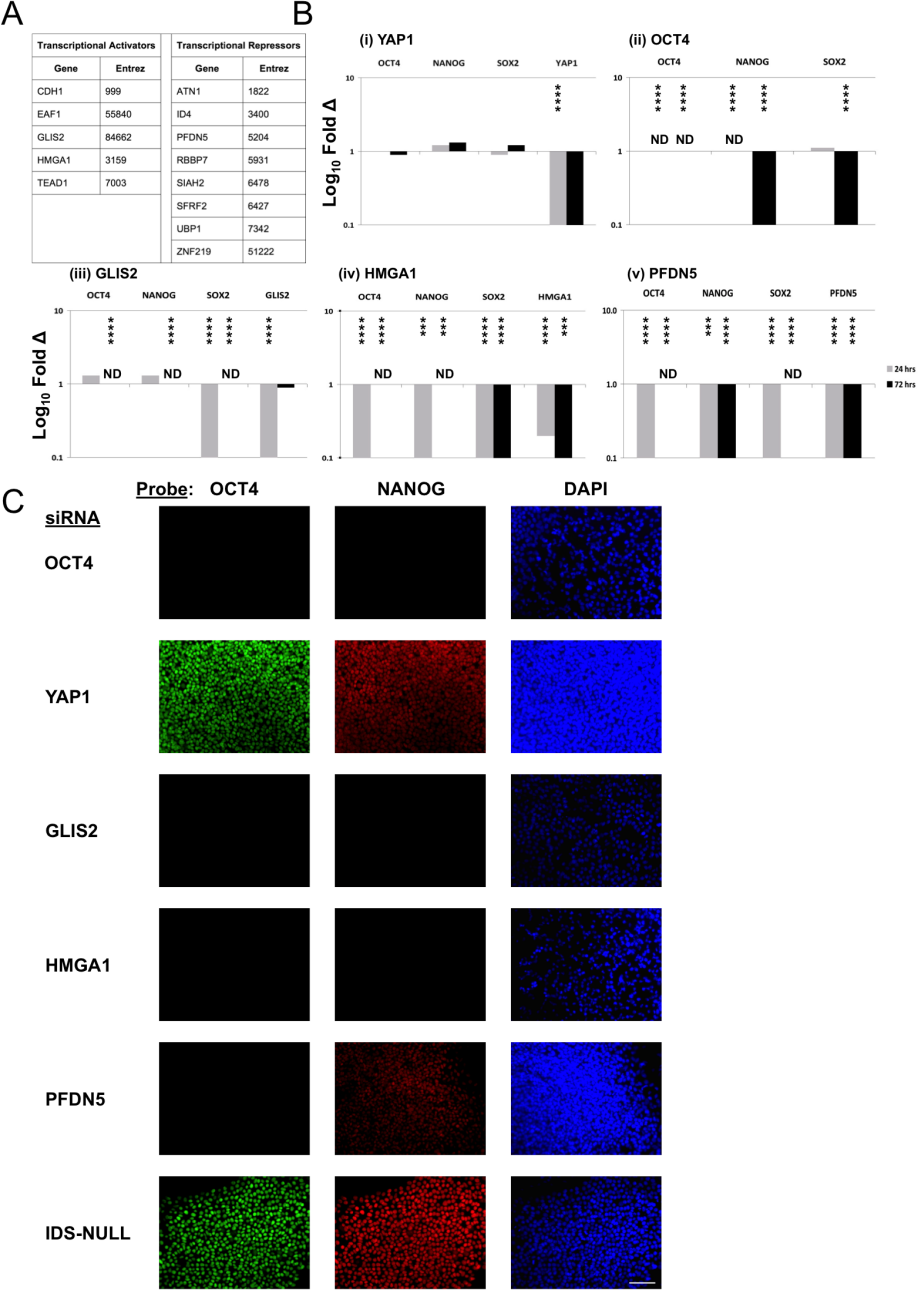
Epigenetically-defined hESC biomarkers have a role in pluripotency. (A) Transcriptional activators (Expressed hESC-UnMe-GA-CGIs) and transcriptional repressors (Expressed hESC-Me-GA-CGIs) identified as functionally overrepresented hESC biomarkers. (B, C) Functional testing of transcriptional regulators in RH1 hESCs by siRNA knockdown. (B) RT-qPCR data showing log_10_ fold change in expression of the siRNA-targeted gene, and associated effects on OCT4, NANOG and SOX2. Changes are relative to GAPDH expression, normalised to RH1 hESCs treated with negative control siRNA IDS-NULL. Asterisks indicate levels of statistical significance (unpaired t-test; *≤0.05, **≤0.01, ***≤0.001, ****≤0.0001). ND: Not Detected, even at 40 cycles of PCR. (C) Immunohistochemistry for NANOG and OCT4 72 hours after siRNA treatment. Scale bar = 100 μm.

Correlation of Me-GA-CGIs and UnMe-GA-CGIs (Table D in S1 File) with transcriptome data identified expressed genes with an hESC-specific CGI methylation state (Tables E-G in S1 File). Of 109 gene loci with an hESC-UnMe-GA-CGI, 78 genes with non-redundant Entrez IDs were expressed. Similarly, of 216 gene loci with an hESC-Me-GA-CGI, 169 unique genes were expressed. The subset of CGI-associated genes may be enriched for functional categories, so we first analysed the distribution of genes associated with CGIs on the array, and identified 11357 non-redundant Entrez IDs, about 2/3 (64.3%) of the genes annotated in Gene Ontology (17673 genes). CGI-associated genes were enriched (e.g. transcription factor, developmental process) or depleted (egg. receptors, signal transducers) for various categories (S7 Fig and Table J in S1 File); thus the functional analysis of expressed hESC-methylated or unmethylated genes was compared with the set of CGI-associated genes on the array. These hESC-specific gene sets were tested for enrichment in Gene Ontology categories relative to the proportion expected for CGI-associated genes. Transcriptional activators (GO:0016563) are significantly overrepresented among genes with an hESC-unmethylated CGI (GO:0016563, P < 0.01, FDR = 0.01; Table K in S1 File); similarly related GO categories including transcriptional regulator activity (GO:0030528), transcription factor binding (GO:0008134), DNA binding (GO:0003677) and sequence-specific DNA binding (GO:0043565) are also overrepresented (Fig 2A). Only two genes associated with transcriptional repressor activity (MSX1 and TBX3) have an hESC-UnMe-CGI. For genes with an associated hESC-Me-CGI, only two categories were enriched (FDR < 0.25): phosphoinositide binding (GO: 0035091) and transcription repressor activity (GO: 0016564); P < 0.001, FDR = 0.137 in both cases; Fig 2A).

We found no association between hESC-specific CGI methylation and genetic imprinting. With respect to the set of hESC-expressed genes whose associated CGI is methylated in hESCs and unmethylated in somatic tissues, there are no imprinted genes included. In the set of hESC-expressed genes whose associated CGI is unmethylated in hESCs and methylated in somatic tissues, there are two imprinted genes, GNAS and SLC22A3, but SLC22A3 is known to be imprinted in a limited fashion, being only monoallelically expressed in the placenta during the first trimester [33]. Comparing our epigenetic biomarkers dataset with the 231 known imprinted genes as a proportion of the most recent estimate of ~19,000 human genes [34], there was thus no significant overlap (χ^2^ = 0.692, P = 0.405, 1 DF).

### Functional Significance of Epigenetically-Defined hESC Biomarkers

**Fig 3:**
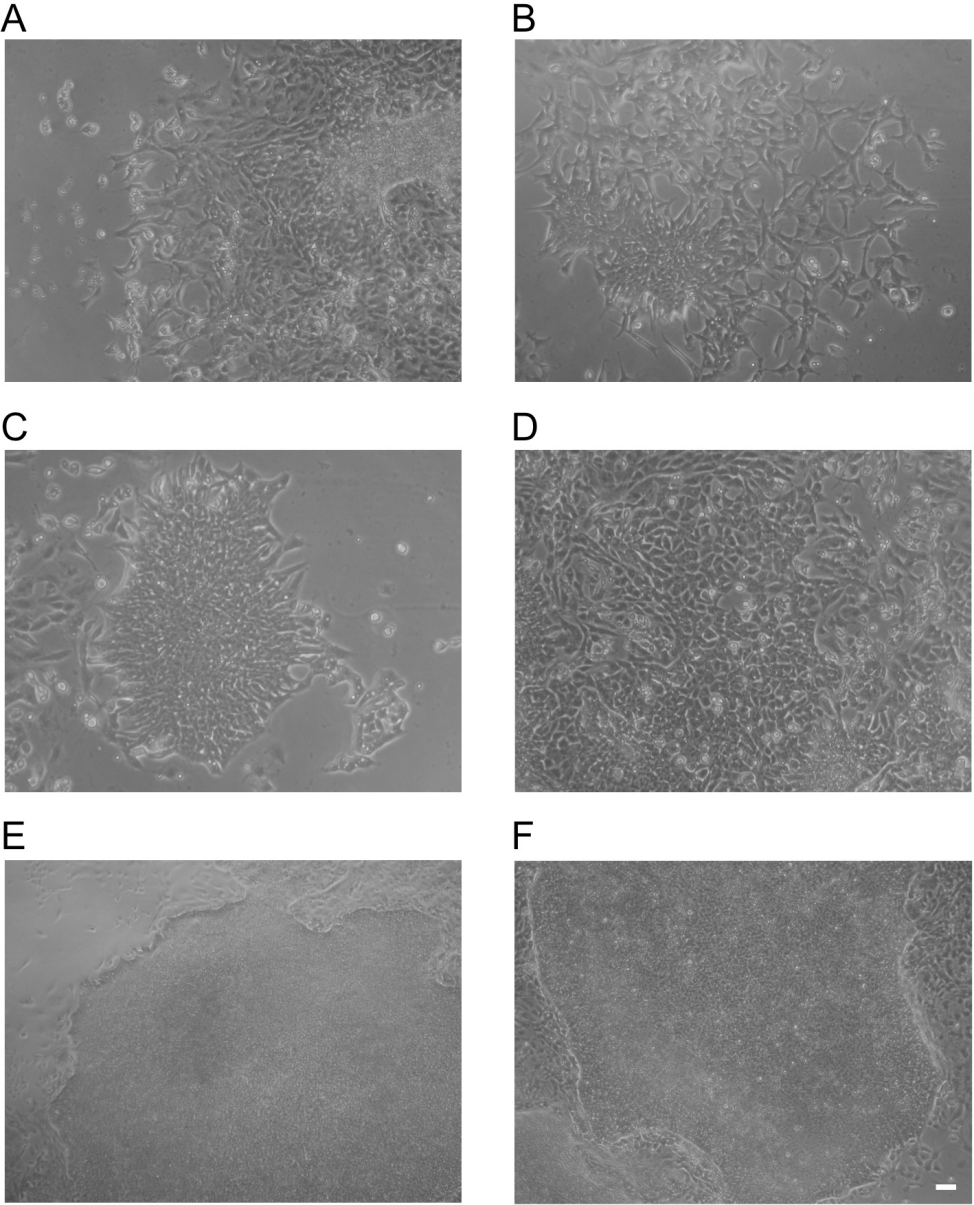
Epigenetically-defined hESC biomarkers are required for stem cell phenotype. Morphological changes in RH1 hESCs knocked down for (A) OCT4 and (B-D) knockdown of GLIS2, HMGA1 and PFDN5, respectively. Normal hESC morphology was maintained with (E) anti-YAP1 or (F) IDS-NULL negative control siRNA treatment. Scale bar = 100 μm.

**Fig 4:**
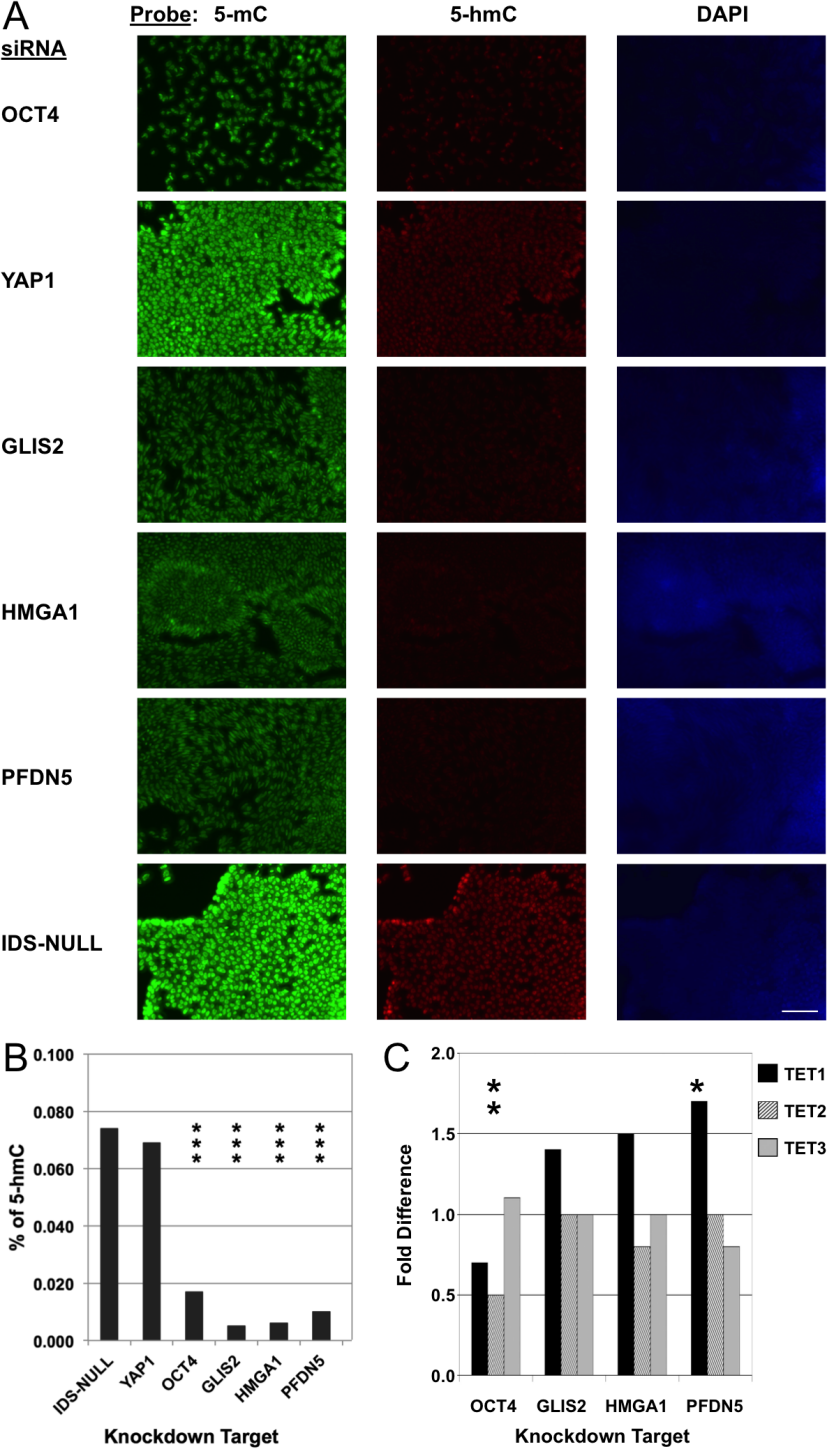
Interference with epigenetically-defined biomarkers perturbs the hESC epigenome. RH1 hESCs were treated with siRNAs as indicated. (A) Immunohistochemical staining for 5-methylcytosine (5-mC) and 5-hydroxymethylcytosine (5-hmC). After knockdown of OCT4, GLIS2, HMGA1 or PFDN5, 5-hmC is difficult to detect. Knockdown of YAP1 or the negative control siRNA (IDS-NULL) had no effect on 5-hmC in hESCs. Scale bar = 100 μm. (B) Quantification of 5-hmC by ELISA as a percentage of total cytosine in gDNA confirming a large (>80%) fall in 5-hmC in hESCs when OCT4, GLIS2, HMGA1 or PFDN5 is knocked down. (C) RT-qPCR data showing fold change in TET1-3 expression on OCT4, GLIS2, HMGA1 or PFDN5 knockdown. TET transcript level changes were mostly not significant or modest (within ~1.5-fold). Asterisks from 1–4 indicate levels of statistical significance, cf. IDS-NULL.

**Fig 5:**
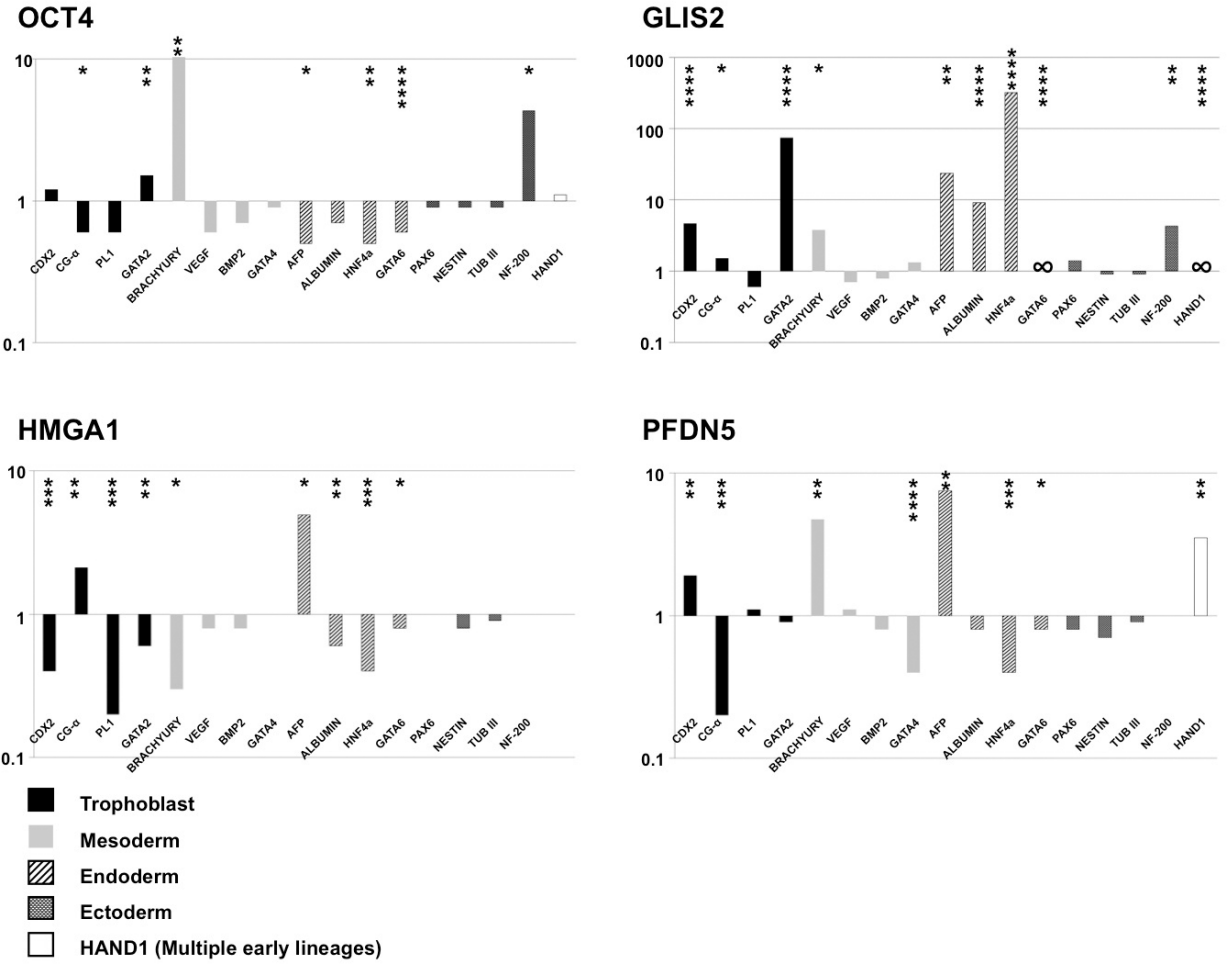
Embryonic lineage preference in hESCs after downregulation of epigenetically-defined biomarkers. Expression at 72 hours after RH1 hESCs were treated with siRNAs indicated. RT-qPCR data showing log_10_ fold change in expression of the indicated embryonic lineage marker gene. Changes are relative to GAPDH expression, normalised to IDS-NULL-treated RH1 cells. Asterisks indicate levels of statistical significance (unpaired t-test; *≤0.05, **≤0.01, ***≤0.001, ****≤0.0001). ND: Not Detected, even at 40 cycles of PCR.

We tested the functional role of three candidate epigenetically-defined hESC biomarkers: the transcriptional activators GLIS2 and HMGA1, and the repressor PFDN5, as all are expressed significantly over background (Table L in S1 File, microarray expression data for GLIS2, HMGA1 and PFDN5). Small Interfering RNA (siRNA) transfection conditions in hESC lines were optimised (S8 Fig). RH1 was selected to represent the cell lines in this study, with results independently confirmed in H9 one of the most commonly-studied hESC lines. HESCs were transfected with Lipofectamine RNAiMAX and siRNA oligonucleotides (Table M in S1 File, sequences of siRNA oligonucleotides used in this study) twice, 24 hours apart, and samples were taken at 48 and 96 hours after the first transfection. An siRNA targeting no human transcript (IDS-NULL, directed against IDS but containing 4 point mutations) was used as a negative control for sample normalisation from the same cell line and time point. An siRNA directed against OCT4 was used as a positive control for effects on pluripotency, and an siRNA directed against YAP1 to control for responses to knockdown of an hESC-expressed gene unnecessary for pluripotency[35]. Response to knockdown of all three genes was similar (RH1, Figs 2–5 and H9, S9–S12 Figs). Target transcript knockdown was significant and strong (typically > 90%) by 24 hours post-siRNA treatment 2 (Fig 2B and S9A Fig). Despite rapid (24 hr.) downregulation of YAP1 to ~10% normal levels, we saw no effect on OCT4, NANOG or SOX2 in RH1 (Fig 2Bi) and minimal effects in H9 (S9Ai Fig). However OCT4 knockdown was accompanied by significant reductions in NANOG and SOX2 (Fig 2Bii and S9Aii Fig). These controls show that our siRNA knockdown system was (1) not “swamping” the cellular RNA degradation system, inducing non-specific effects that perturbed normal phenotype, (2) efficient, producing a measurable target knockdown, and (3) yielded expected effects when targeting genes of known function.

HMGA1, GLIS2 and PFDN5 were significantly downregulated by siRNA treatment (Fig 2Biii-v and S9Aiii-v Fig). In RH1 cells HMGA1 and PFDN5 expression remained downregulated (~10% normal transcript levels) at 72 hours, but GLIS2 transcripts recovered to approximately normal levels by this time (Fig 2Biii-v and S9Aiii-v Fig). In H9 cells, GLIS2 and PFDN5 knockdowns were very efficient, HMGA1 less so, appearing to recover and "overshoot" normal transcript levels by 72 hrs. (S9Aiii-v Fig). Whenever GLIS2, HMGA1 or PFDN5 expression was knocked down, OCT4, NANOG and SOX2 were also downregulated with respect to controls (Fig 2Aiii-v and S9Aiii-v Fig).

Immunostaining of anti-OCT4 siRNA-treated hESCs showed no OCT4 signal, as expected, or for NANOG, consistent with RT-qPCR data showing NANOG falling on OCT4 knockdown (Fig 2C and S9B Fig). Both, however, were readily detectable in cells treated with either anti-YAP1 siRNA or IDS-NULL (Fig 2C and S9B Fig). Anti-HMGA1,-GLIS2 and-PFDN5 siRNA-treated hESCs showed no detectable OCT4 signal or, in the cases of GLIS2 and HMGA1, NANOG signal. Anti-PFDN5 siRNA-treated hESCs did show a detectable NANOG signal, but noticeably weaker than negative controls (Fig 2C and S9B Fig).

OCT4, NANOG and SOX2 downregulation induced by siRNA to OCT4 or HMGA1, GLIS2, or PFDN5 was accompanied by morphology changes consistent with differentiation. In contrast, siRNAs anti-YAP1 and IDS-NULL induced no such changes (Fig 3 and S10 Fig). As stem cell differentiation is accompanied by loss of genomic DNA hydroxymethylation (Ruzov et al., 2011) we evaluated effects on this epigenetic mark and on expression of TET dioxygenases (TET1, 2, 3). Compared with anti-YAP1 and IDS-NULL, knockdown of OCT4, HMGA1, GLIS2 and PFDN5 reduced genomic levels of 5-hmC in both lines, as shown by quantitative ELISA (Fig 4B and S11B Fig; P<0.001). Consistent with these results, the immunofluorescence-detectable signal for 5-hmC in both cell lines was attenuated (Fig 4A and S11A Fig). This reduction in 5-hmC levels could not be attributed to a reduction in TET expression as transcript levels were either unchanged or only modestly so (< 2-fold; Fig 4C and S11C Fig). In some cases, most notably with the RH1 cell line (Fig 4), there was also some apparent reduction in global mC levels detectable by immunofluorescence, but it should be noted that the same effect was observed with OCT4 as well as with HMGA1, GLIS2 and PFDN5.

To determine whether GLIS2, HMGA1 and PFDN5 knockdown resulted in undirected differentiation or biased lineage selection, we quantified lineage-associated markers by RT-qPCR. In RH1 cells, OCT4 downregulation caused small reductions of most lineage markers examined, except upregulation of the mesodermal marker Brachyury and the ectodermal marker NF200 (Fig 5). H9 cells responded differently, upregulating AFP and other trophoblast and endodermal markers including CDX2, CGα and GATA4, consistent with previous data (S12 Fig, [36]). GLIS2 downregulation induced changes in several genes, including upregulation of the endodermal markers α-fetoprotein (AFP), albumin and either HNF4α (RH1) or GATA6 (H9) (Fig 5 and S12 Fig). Trophoblast markers were also upregulated in both lines on GLIS2 knockdown (Fig 5 and S12 Fig). While AFP was also upregulated in cells of both lines knocked down for HMGA1, most endodermal and trophoblast markers other than CGα (upregulated) were downregulated. PFDN5 knockdown also induced AFP upregulation but, as with HMGA1, other endodermal markers tended to be downregulated or unchanged. In RH1 cells, PFDN5 also resulted in upregulation of HAND1 (expressed by multiple early lineages) and Brachyury (mesendoderm), but H9 differed; here both were downregulated. Overall therefore, there were similarities in response, but even the OCT4 knockdown response was not uniform between lines. GLIS2 knockdown gave the most consistent response between cell lines.

**Fig 6:**
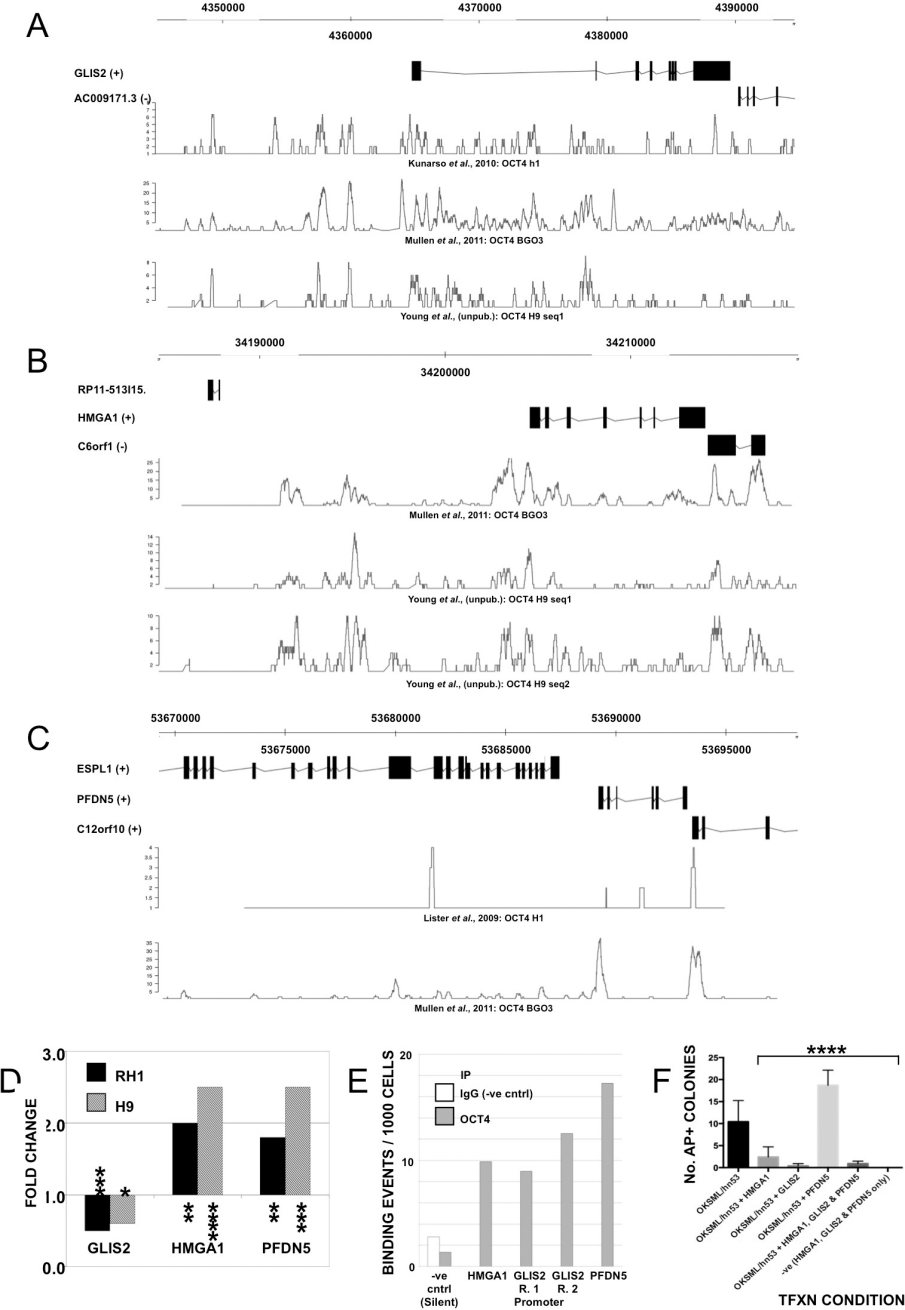
Association of candidate epigenetically-defined biomarkers with the core pluripotency network. GLIS2 (A), HMGA1 (B) and PFDN5 (C) all have OCT4 binding sites in their promoter regions. Panels created by Geneprof (geneprof.org) using OCT4 ChIP-seq data from Young and colleagues (unpublished), Kunarso *et al*. (2010) and Mullen *et al*. (2011) (GLIS2), Young and colleagues (unpublished) and Mullen *et al*. (2011) (HMGA1) and Lister *et al*. (2009) and Mullen *et al*. (2011) (PFDN5); Y-axis indicates read number from the dataset indicated. (D) OCT4 knockdown in hESCs as described perturbs HMGA1, GLIS2 and PFDN5 transcript levels. Asterisks indicate levels of statistical significance (unpaired t-test; *≤0.05, **≤0.01, ***≤0.001, ****≤0.0001). (E) Chromatin immunoprecipitation (ChIP)-qPCR suggests that HMGA1, GLIS2 and PFDN5 expression is regulated by OCT4. Immunoprecipitation of hESC chromatin with an antibody to OCT4, followed by quantitative amplification of gene-specific promoter regions above background (a silent region of the genome) in hESCs. (F) Representative experiment whereby human dermal fibroblasts (HDFs) were transfected with a set of three episomal plasmids expressing OCT4, SOX2, KLF4, L-MYC, LIN28 and a short hairpin RNA directed against p53 (OKSML/hn53) alone (left-most column) or with OKSML/hn53 supplemented with either HMGA1, GLIS2 or PFDN5, or all three, followed by detection of alkaline phosphatase positive colonies (No. AP+ Colonies) representative of pluripotent cells. Error bars indicate standard deviation of the mean; **** indicates the level of statistical significance (P≤0.0001).

As perturbation of either OCT4 or HMGA1, GLIS2 or PFDN5 (all transcriptional regulators) results in loss of stem cell phenotype, and biomarker knockdown also results in downregulation of OCT4, NANOG and SOX2, a transcriptional connection between the core pluripotency factors and biomarkers is possible. GLIS2, HMGA1 and PFDN5 all apparently contain OCT4 binding sites in their promoters (Fig 6A–6C; [37, 38] R. Young, Whitehead Institute, Cambridge, MA [unpublished dataset], and [25], and also binding sites for other transcription factors which interact with OCT4, NANOG and SOX2, including EOMES, TRIM28, E2F1, CDX2 and ETS1.

OCT4 knockdown in hESCs perturbed GLIS2, HMGA1 and PFDN5 expression (Fig 6D), but although statistically significant, the mRNA changes were smaller than those seen for OCT4, NANOG and SOX2 in response to GLIS2, HMGA1 or PFDN5 knockdown, where the pluripotency factors were rapidly and completely repressed. OCT4 knockdown downregulated GLIS2 by ~2-fold, and upregulated HMGA1 and PFDN5 by ~2.5-fold in both lines (Fig 6D). To confirm the proposed link between the epigenetic biomarkers and core pluripotency network, we performed ChIP-qPCR for transcription factor promoter binding in hESCs (Fig 6E). We used *Matinspector* (Genomatix software; [39]) to predict OCT4, SOX2 and NANOG binding sites in the promoters of HMGA1, PFDN5 and GLIS2, and similarly HMGA1 and GLIS2 binding sites in the promoters of OCT4, SOX2 and NANOG. Amplification of HMGA1, GLIS2 and PFDN5 promoter regions from cross-linked, sheared chromatin immunoprecipitated with an antibody to OCT4 indicated that OCT4 is DNA-bound at the predicted loci in hESCs (Fig 6E). We were unable to identify antibodies for HMGA1 and GLIS2 suitable for chromatin-immunoprecipitation to confirm their binding to the promoters of OCT4, SOX2 and NANOG.

### Ectopic Expression of Epigenetic Biomarkers in Differentiated Cells

Unlike the pluripotency factors OCT4 and NANOG, expression of HMGA1, GLIS2 and PFDN5 is not limited to stem cells. To identify a role in conferring pluripotency as well as its maintenance, we reprogrammed human dermal fibroblasts by transfection with episomal plasmids expressing OCT4, KLF4, SOX2, L-MYC, LIN28 and a short hairpin RNA directed against p53 [32], and added similar plasmids expressing HMGA1, GLIS2 or PFDN5 (S13 Fig). In 4 experiments supplementing the basic reprogramming set with HMGA1, GLIS2, or HMGA1, GLIS2 and PFDN5 together, the epigenetically-identified factors either had no significant effect on the number of colonies obtained that were positive for the early pluripotency reprogramming and stem cell marker alkaline phosphatase, or reduced it. This effect was more noticeable with GLIS2 than HMGA1. PFDN5 however, apparently increased AP+ colonies, either significantly (Fig 6F) or not where greater variability in colony numbers between treatment replicates was seen, but the general trend was seen in all experiments (Fig 6F and S14 Fig).

## Discussion

We have defined a CGI methylation map specific to hESCs. Whilst overall CGI methylation in hESCs is similar to adult tissues, gene-associated CGI methylation is reduced, consistent with the view that pluripotent cells possess an open chromatin structure permissive of gene expression (Fussner et al., 2010; Gaspar-Maia et al., 2011; Meshorer et al., 2006). Gene association of CGIs (within 1.5kb of or overlapping an annotated gene) included “orphan” CGIs of uncertain significance. Such CGIs may indicate novel promoters for alternative transcripts or non-coding RNAs regulating gene expression [40, 41]. Expression of a gene associated with an Me-CGI could reflect suppression of an alternative transcript or non-coding RNA, detailed sequence based maps for which now exist for hESCs [42, 43].

As in other hESC DNA methylation studies [14–17, 44], we saw substantial variation in CGI methylation between lines, probably related to differences in line provenance, cultivation method and passage number. This variation was particularly evident on the X-chromosome, probably reflecting the 3 classes of X inactivation status seen in hESC lines (See supplementary discussion A in S1 Document).

Cross-referencing of the CGI methylation map to the transcriptome yielded a panel of 184 hESC-expressed genes as putative epigenetically-defined biomarkers of a pluripotent phenotype (S15 Fig). Expressed genes whose associated CGI was methylated in hESCs were unbiasedly distributed throughout the genome, but those whose CGI was unmethylated were over-represented on chromosome 16. These included GLIS2 (16p13.3), and Cadherin Type 1 (16q22.1), a recognised pluripotency factor [45], and seven other genes implicated in signal transduction and chromatin remodelling which have not been assigned roles in pluripotency.

HESC epigenetically-defined biomarkers were significantly enriched for transcriptional control functions, generally transcriptional activators for those associated with UnMe-CGIs, and repressors for those associated with Me-CGIs. We chose the transcriptional activators GLIS2 and HMGA1, and the repressor PFDN5 as candidates to test for a role in pluripotency. GLIS2 encodes a zinc finger transcription factor which interacts with p120 catenin (a member of the pluripotency-associated WNT pathway); it is expressed modestly in hESCs (Table L in S1 File). The GLIS2 homologue GLIS1 promotes reprogramming of fibroblasts to iPS cells [46]. HMGA1 has been implicated in tumour development [47] and recently, but unknown at the time our study was initiated, as a reprogramming factor [48]. It is known to interact with molecules influencing hESC phenotype including Lin28, WNT, MYC, STAT3 and GSK3 and its transcription is modulated by KLF4 and OCT family members. PFDN5 is a molecular chaperone highly expressed in hESCs (Table L in S1 File) and interacts with both MYC and WNT, genes with important roles in growth and self renewal of ECSs [49]. Collectively, these interactions are consistent with our findings that interference with these genes initiates differentiation. Downregulation of candidate biomarkers resulted in variable effects on the expression of specific lineage-associated markers. GLIS2 is associated with both neural (ectodermal) and kidney (mesodermal) development [50]. Downregulation of GLIS2 upregulated multiple markers of endodermal and extraembryonic lineages, consistent with these roles. HMGA1 and PFDN5 knockdown also upregulated endoderm-associated AFP, but effects on other endodermal and extraembryonic markers were less pronounced or inconsistent. However, we only assessed lineage marker abundance at one timepoint, and differences may be due to variation in interference kinetics or cell response, perhaps in turn related to epigenomic differences between lines.

### Interaction of Epigenetic Biomarkers with the Core Pluripotency Network

**Fig 7:**
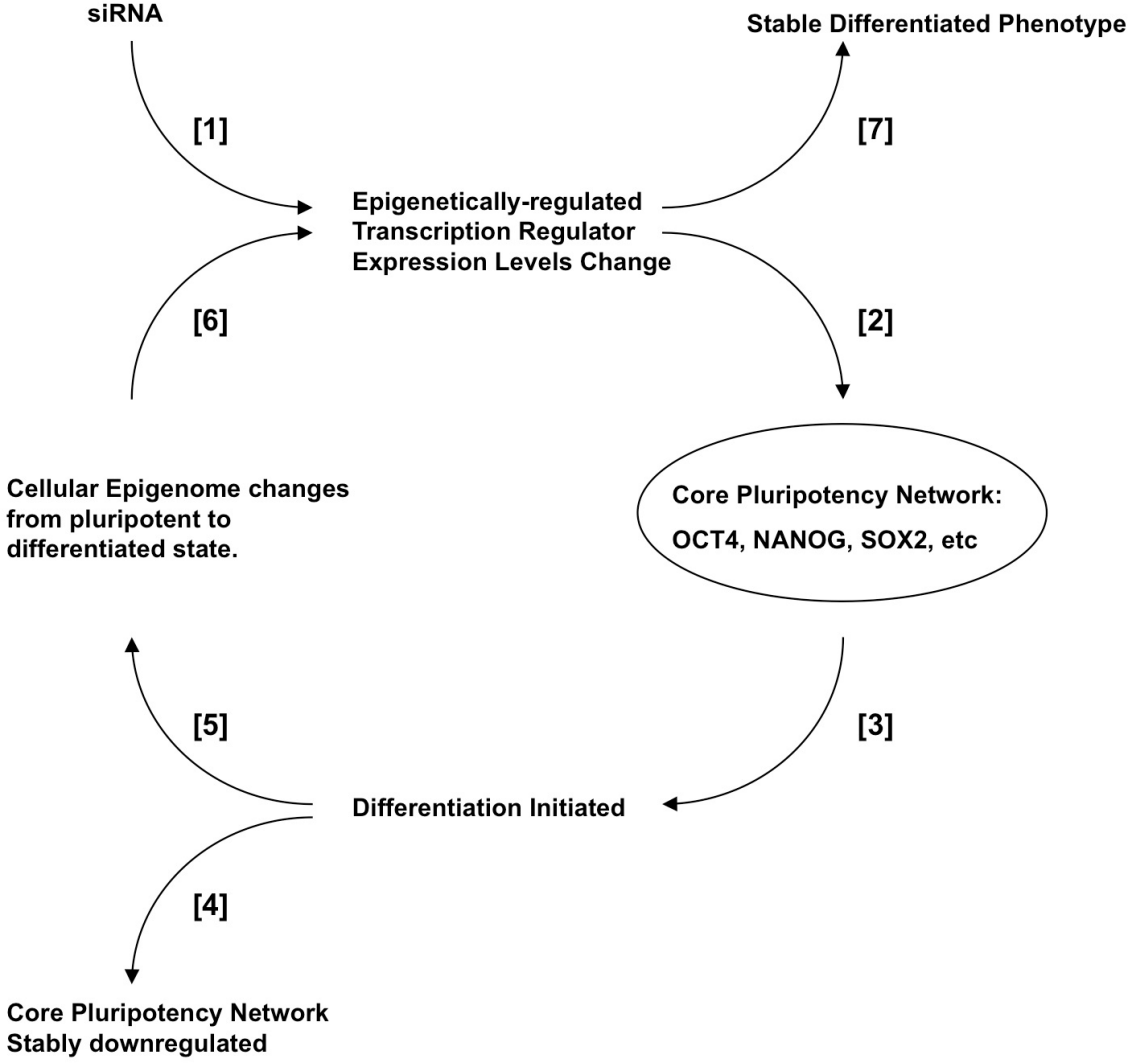
Model of the relationship between epigenetically-regulated hESC biomarkers and the pluripotency transcription system. Changes in the expression of an epigenetically-regulated transcriptional regulator (e.g. HMGA1, GLIS2, PFDN5) achieved by siRNA transfection [1], or changes in associated CGI methylation [6], feed through the cellular transcription network, resulting in a reduction in core pluripotency transcription factors (OCT4, NANOG, SOX2, [2]) and differentiation [3]. The core pluripotency transcription factors are permanently downregulated [4], and epigenetic changes to gene promoters, CGIs and other regulatory regions of the genome occur to confer stability on the differentiated phenotype and prevent reversion to pluripotency or quasi-pluripotency [5]. These changes confer permanent changes to the expression of the epigenetically regulated transcriptional regulators [6], and thus stabilise the differentiated phenotype [7].

We confirm a role for GLIS2, HMGA1 and PFDN5, and by inference other genes with an hESC-specific CGI-methylation state, in the maintenance of pluripotency transcription factor expression and pluripotency-associated epigenetic marking (DNA hydroxymethylation). For GLIS2 and PFDN5, and some functions of HMGA1, these roles are novel and suggestive of an epigenetically-defined network of stem cell regulation by genes also expressed in some differentiated cells. We surmised that this network would be controlled in turn by pluripotency-determining factors, and indeed ChIP confirmed the existence of predicted OCT4 binding sites in the promoters of all three genes [25, 37, 38]. Probable binding sites for NANOG and/or SOX2 in the biomarker gene promoters were also identified (data not shown). Our observation of a modulating effect on fibroblast reprogramming (positive for PFDN5, negative for HMGA1 and GLIS2) transfected with established reprogramming factors is consistent with the hypothesis that the biomarkers interact with the core pluripotency factors at some level. The inhibitory effects of HMGA1 and GLIS2 may reflect time- or phase-dependent roles for these factors in reprogramming which our experimental design did not address, as recently described for MBD3 [51], or reflect competing interaction with other factors. HMGA1-induced augmentation of reprogramming was achieved by co-transfection with OCT4, KLF4, SOX2 and L-MYC [48], whereas our study also included LIN28 and a short hairpin RNA directed against p53. Further studies are required to confirm interactions of GLIS2 and PFDN5 with pluripotency transcription factors. Binding sites for GLIS2 are predicted in the promoters of OCT4 and NANOG (data not shown). PFDN5 is known to repress c-MYC activity which regulates genes involved in many processes including cell-cycle control, metabolism, signal transduction, and cell-fate decisions as well as self-renewal (Chappell and Dalton, 2013). HMGA1 binding sites are predicted in the promoters of OCT4 and NANOG (data not shown) and have been shown by chromatin immunoprecipitation in SOX2, LIN28 and c-MYC [48]. The reduction in DNA hydroxymethylation, not accompanied by similar falls in TET gene transcription, following interference with HMGA1, GLIS2, or PFDN5 suggests that these effects are secondary events following disruption of core pluripotency functions. Collectively, our data suggest a model as shown in Fig 7. Downregulation of biomarker expression, either experimentally by RNA interference, or by methylation changes at associated CGIs modulating gene expression, results in downregulation of the core pluripotency transcription network, directly or indirectly. Differentiation is initiated, with the particular lineage decision decided by the presence or absence of secondary transcription regulators, such as epigenetically-regulated factors identified here. Stability of the differentiation process is conferred both by downregulation of pluripotency genes (NANOG, SOX2 and OCT4), and also by epigenetic changes, e.g. OCT4 promoter [52, 53], or methylation changes in CGIs associated with particular lineages. The methylation changes confer stability and heritability on the gene expression changes, and hence on the resulting cellular phenotype.

In conclusion, we identify epigenetically defined biomarkers of a pluripotent phenotype. The methylation state of these biomarkers is independent of variables such as culture condition or derivation method, and their expression is required for pluripotency. Appraisal of the methylation state of the CGIs described here could be a useful criterion for hESC line validation, or for assessing how well adult cell-derived IPSCs have established a true embryonic stem cell epigenetic state.

## Supporting Information

S1 Document Supplementary Discussion. (A) X-inactivation status of female hESC lines RH1, RH3 and RCM1. (B) The relationship between expression profile and genomic methylation, and cell line derivation and culture conditions.
(DOCX)

S1 File Supporting Information Tables. (A). Summary of hESC lines employed in this study. (B) Methylation of X-linked gene-associated CGIs in hESC Lines. (C) CGIs removed from consideration as methylated in all hESC lines because of inconsistent behaviour between multiple reporters. (D) hESC-specific gene associated methylation. (E) Correlation of hESC-specific CGI methylation status and transcriptome. (F) Expressed genes with an associated CGI which is always methylated in hESCs and always unmethylated in somatic tissues. (G) Expressed genes with an associated CGI which is always unmethylated in hESCs and always methylated in somatic tissues. (H) Assessment of differential CGI methylation and differential gene expression between female hES lines. (I) Differentially methylated and expressed genes are strongly associated with the X chromosome in female hESC lines. (J) Enrichment and depletion of functional categories in Gene Ontology of genes with an associated CGI in the human genome. (K) Summary of GO analysis indicating over-represented functions of epigenetically defined biomarkers of hESCs. (L) Microarray probe data for selected candidate epigenetic biomarkers GLIS2, HMGA1 and PFDN5 in female hESC lines. (M) Sequences of siRNAs employed in this study. (N) Microarray probe data for *XIST* expression in female hESC lines. (O) Primers used for RT-qPCR analysis. (P) Primer sequences for amplification of indicated gene promoter region after chromatin immunoprecipitation for OCT4 in hESCs. (Q) Table listing unmethylated CGIs in hESCs and somatic tissues.
(DOCX)

S1 Fig Schematic depicting the isolation of methylated genomic DNA from human ES cells, column purification of methylcytosine-rich sequences (i.e., methylated CGIs) and input and MAP-purified DNA hybridisation to a custom array of 17,000 CGIs. CGI Array Notes: CGI sequences on this array were identified on the basis of column-based binding of *MseI*-cloven genomic DNA from human blood mononuclear cells (pooled from 3 male donors) to a recombinant cysteine-rich CXXC3 domain of mouse Mbd1, which is characterised by a high affinity for non-methylated CpG sites (Voo et al., 2000; Jorgensen et al., 2004). *MseI* cleaves the sequence TTAA, and as such normal gDNA is cloven into small fragments (predicted average size 125 bp) containing typically 1 or 2 CpG dinucleotides. TTAA sites are underrepresented in CGIs, resulting in CGI-derived fragments of an average size of ~625 bp, and containing typically 50–60 unmethylated CpGs, enabling purification and subsequent sequencing of CGI-containing fragments. Because of Mbd1’s non-methylated CGI affinity based-purification, the resulting array excluded the small fraction of CGIs that are fully methylated in somatic cells, estimated to be less than 3% (Weber et al., 2007). Large scale sequencing of the column-bound fraction identified both CGIs predicted by CGI prediction algorithms and annotated in the ENSEMBL database, but also many CGIs that were predicted and only identified by their interaction with the mbd1 domain. As such, the CGIs on the array are biologically defined rather than defined by an algorithm.
(TIF)

S2 Fig Genome-wide CGI hybridisation analysis. Chromosomes are ordered 1–22, X (23), Y (24), top to bottom. Each vertical tick mark represents an annotated CGI. Blue indicates <0.5 (log2) hybridisation in RH4 (male hESC line) with respect to RH3 (female hESC line) (i.e. reduced DNA hybridisation), yellow indicates broadly similar hybridisation (0.5 [log2] ≥ qty ≤1.5[log2]) and red indicates greater hybridisation in RH4 than RH3 (>1.5 [log2]). RH3 c.f. RH4 gDNA input control comparison shows globally similar levels of hybridisation (yellow) throughout the genome, as expected for total DNA from two euploid human cells lines, with the exceptions of (1) X chromosome hemizygosity and hence reduced signal (blue) in RH4 (male line), with the exception (2) of the pseudo-autosomal region PAR1 (close to the telomere of the short arm [left hand end of chromosome 23]). PAR2 is close to the telomere of the long arm of the X chromosome, but is smaller (320 kb as opposed to 2.6 Mb for PAR1) with fewer mapped CGIs, and hence less visible, and (3) Increased hybridisation (red) in the Y chromosome (24).
(TIF)

S3 Fig Bisulphite Sequencing of hESC CGIs confirms CGI methylation array data. (A) Details of two X-linked gene-associated CGIs used for verification of array-defined CGI methylation status and relationship of gene expression. (B) CGI I24453 (associated with SCML1) is confirmed as differentially methylated, being methylated on one allele in RH1 and both alleles being unmethylated in RH3, as indicated in the CGI array data, whereas (C) CGI I24952 (associated with IDS) is unmethylated in both cell lines. In all examples, black circles indicated methylated CpGs, and white circles indicate unmethylated CpGs. (D) Affymetrix U133Plus2 genechip probe data (Log2 probe signal for RMA-normalised data; three independent replicates for each cell line) for SCML1 and IDS. (Entrez references 6322 and 3423 respectively) show expression in female hESC lines RH1 and RH3. SCML1 is expressed at approximately 1.5-fold higher levels in RH3 compared to RH1, consistent with expression from both alleles in RH3 and one allele in RH1. IDS is expressed at approximately equal levels in both cell lines (RH3 ~1.18 fold higher levels than RH1). All fold changes are given as Log2.
(TIF)

S4 Fig Heterogeneity of CGI methylation between hESC Lines. In all panels, chromosomes are ordered 1–22, X, Y, top to bottom. Each vertical mark represents an annotated CGI. Blue indicates <0.5 (log2) hybridisation compared with the other line (i.e. reduced CGI methylation), yellow indicates broadly similar hybridisation (0.5 [log2] ≥ qty ≤1.5[log2]) and red indicates greater binding/methylation (>1.5 [log2]). (A) RH1 c.f. RCM1 MAP- gDNA shows heterogeneity between the two lines, similarly (B) between RH3 and RCM1, indicating heterogeneity in levels of CGI methylation between both pairs of cell lines. (C) RH1 c.f. RH3 shows these two lines are more similar to each other in their CGI methylation than to RCM1 throughout the genome, but that RH1 has higher levels of CGI methylation on the X chromosome (line 23, red ticks frequent). (D) Comparison of X chromosome hybridisations indicating similar total input gDNA content for the X chromosome in the two female lines RH1 and RH3 (i.e., both are euploid female lines with a 46XX karyotype) (i), but over-representation of methylated CGIs in RH1 compared with RH3 when the MAP-gDNA is examined (ii, see also C)
(TIF)

S5 Fig Summary of Affymetrix U133Plus2 Genechip analysis of hESC Lines’ transcriptome. (A) Summary indexes (probe set expression levels) after RMA processing including quantile normalisation show very similar distributions for all nine arrays. (B) Scatter plots of expression values between different arrays of the same cell line show a high correlation (Pearson correlation coefficient r>0.99), and thus (C) the arrays cluster according to biological samples of the three cell lines. (D) Codes for the three replicates of each cell line.
(TIF)

S6 Fig X-Linked gene expression in female hESC lines suggests X inactivation in RH1 and RCM1, but not RH3. (A) Annotated X-linked probe relative expression level for RMA normalised data along the length of the X chromosome, comparing the pairs of cell lines indicated, shows broadly similar expression levels for RH1 and RCM1 (top panel; yellow tick indicates Signal_RCM1_ ≥ 0.75 x Signal_RH1_ and ≤ 1.5 x Signal_RH1_; red tick indicates Signal_RH1_ ≥ 1.5 x Signal_RCM1_ and blue tick indicates Signal_RH1_ < 0.75 x Signal_RCM1_). However, RH1 X-linked genes show frequently lower levels of expression when compared with RH3 (middle panel; blue ticks), with the exception of the pseudoautosomal region 1 located at Xp22 (left hand end, yellow ticks). Similarly, when RH3 is compared with RCM1 (lower panel), X-linked gene expression in RH3 is typically > 1.5-fold the levels observed in RCM1 (red ticks). Few significant biologically-relevant differences in expression of genes in the pseudoautosomal regions (B) PAR1 or (C) PAR2 were observed between the female hESC lines RH1, RH3, and RCM1. *N* = 3 independent replicates for each probe, plots indicate Log2 signal ± SD.
(TIF)

S7 Fig Gene Expression in hES cells. Expression threshold was set using probe set present/absent calls generate by the MAS5 algorithm (*Affy* R package). Calls were averaged with respect to corresponding Entrez Gene ID and subsequently over replicate samples. Genes were designated as expressed if average P > 0.5. Overlap of the genes expressed in RH1 (11902), RH3 (12004) and RCM1 (11742) showed most genes were expressed in all three lines, as expected. See also Table F in S1 File.
(TIF)

S8 Fig Optimisation of siRNA transfection in hES Cells. Lipofectamine RNAiMAX (Invitrogen), JetPRIME (Polyplus), INTERFERin (Polyplus) and Safectin (Deliverics, in the presence of either mTeSR1 hESC medium, or Optimem low-serum transfection medium) were compared for their ability to generate red channel-positive cells when used to transfect RH1 hESCs with a Cy3-labelled negative control siRNA directed against no human transcript (IDS-NULL). (A) Lipofectamine RNAiMAX showed the highest rates of transfection of the four reagents tested. No significant difference in the proportion of positive cells was observed between INTERFERin and JetPRIME or between Lipofectamine RNAiMAX and JetPRIME; nor did the medium in which Safectin was employed affect the transfection efficiency. (B, C) With Lipofectamine RNAiMAX-mediated siRNA transfection, siRNA quantity (B) but not the volume of transfection reagent used (C) affected transfection efficiency, summarised in (D) ANOVA results table.
(TIF)

S9 Fig Epigenetically-defined hESC biomarkers have a role in maintenance of pluripotency. H9 hESCs were treated with siRNAs directed against the mRNAs indicated, with two treatments of siRNA at 0 and 24 hours. Samples were taken at 48 and 96 hours for gene expression analysis (24 and 72 hours after the final siRNA treatment). (A) RT-qPCR data showing log_10_ fold change in expression of the targeted gene, and associated effects on the stem cell transcription factors OCT4, NANOG and SOX2. Changes are relative to GAPDH expression, normalised to H9 hESCs treated with an siRNA which does not target any gene in the human genome (IDS-NULL). Asterisks indicate levels of statistical significance, as calculated by unpaired t-test (*≤0.05, **≤0.01, ***≤0.001, ****≤0.0001). ND: Not detected by 40 cycles of PCR. (i) Knockdown of YAP1, a gene expressed in hESCs but not required for a pluripotent phenotype was successful, but as expected had a minimal effect on the pluripotency markers OCT4, NANOG and SOX2. (ii) Knockdown of OCT4 also results in significant downregulation of associated pluripotency transcripts (NANOG and SOX2). (iii-v) In all cases where transcript levels of an epigenetically-defined biomarker was knocked down, (∞ indicates "infinite KD", i.e., no target transcript was detectable), the knockdown was accompanied by significant reductions in levels of the hESC markers OCT4, NANOG and SOX2. (B) Immunohistochemistry for the hESC markers NANOG and OCT4 at 72 hours after treatment with siRNAs directed against epigenetically defined biomarkers showed that protein levels of the pluripotency markers OCT4 and NANOG are downregulated in association with the reduction in mRNA transcript levels of GLIS2, HMGA1 or PFDN5. Scale bar = 100 μm.
(TIF)

S10 Fig Epigenetically-defined hESC biomarkers have a role in maintenance of the stem cell phenotype. H9 hESCs were treated with siRNAs directed against the mRNAs indicated, with two treatments of siRNA 24 hours apart. Morphology changes were observed in hESCs post-siRNA treatment, with (A) OCT4 knockdown (pluripotency factor) and (B-D) with the epigenetically-defined biomarkers GLIS2, HMGA1 and PFDN5, respectively, but a normal hESC morphology is maintained if either a gene not required to maintain pluripotency is knocked down (E, YAP1), or if a mutant oligonucleotide which does not target any transcripts in the human genome is used (F, IDS-NULL). Scale bar = 100 μm.
(TIF)

S11 Fig Interference with transcription of epigenetically-defined biomarker genes perturbs the hESC epigenome at a global level. H9 hESCs were treated with siRNAs directed against the mRNAs indicated, with two treatments of siRNA 24 hours apart as described previously. (A) Immunohistochemical staining for the epigenetic marks 5-methylcytosine (5-mC) and 5-hydroxymethylcytosine (5-hmC) showed that after knockdown of OCT4, GLIS2, HMGA1 or PFDN5, the stem cell-associated mark 5-hmC becomes more difficult to detect. However, knockdown of either YAP1 or a negative control oligonucleotide (IDS-NULL) had no effect on 5-hmC in hESCs. Scale bar = 100 μm. (B) Quantification of 5-hmC levels by ELISA in cells as a percentage of total cytosine residues in genomic DNA confirms the result of immunochemical staining of 5-hmC, showing a large (>80%), statistically significant reduction in 5-hmC levels in H9 hESCs in which either OCT4 or one of the three epigenetically-defined biomarkers expression is knocked down by siRNA. (C) RT-qPCR data showing fold change in expression of the three TET oxidases in response to knockdown of OCT4 and the epigenetically defined biomarkers GLIS2, HMGA1 and PFDN5. Changes in TET enzyme transcript levels were mostly not significant or modest (within ~1.5-fold) in response to knockdown by OCT4, GLIS2, HMGA1 and PFDN5. Asterisks from 1–4 indicate levels of statistical significance calculated by ANOVA with Dunnett’s post-hoc test, in comparison to the IDS-NULL control.
(TIF)

S12 Fig Embryonic lineage preference in hESCs after downregulation of epigenetically-defined biomarkers of the pluripotent state. H9 hESCs were treated with siRNAs directed against the mRNAs indicated, with two treatments of siRNA at 0 and 24 hours and samples were taken at 72 hours for gene expression analysis (48 hours after the final siRNA treatment). RT-qPCR data showing log_10_ fold change in expression of the indicated embryonic lineage marker gene. Changes are relative to GAPDH expression, normalised to H9 hESCs treated with an siRNA which does not target any gene in the human genome (IDS-NULL). Asterisks indicate levels of statistical significance, as calculated by unpaired t-test (*0.05, **≤0.01, ***≤0.001, ****≤0.0001). ND: Not detected by 40 cycles of PCR. As hESCs initiate differentiation, multiple markers of different embryonic germ layers are up- or downregulated as the cell commits to a particular lineage. Knockdown of GLIS2 upregulated endodermal markers and trophoblast markers. HMGA1 knockdown produced a mixed response with respect to trophoblast markers (extraembryonic tissues) and endodermal markers, but also some ectodermal markers were upregulated. PFDN5 knockdown still perturbed lineage marker expression, but in a non-uniform manner, not apparently favouring any one particular lineage over others, with the exception of the ectodermal markers tested, which were not significantly affected. In contrast to the response shown by RH1 hESCs, the multi-early lineage marker HAND1 was significantly downregulated in H9 cells subjected to PFDN5 knockdown.
(TIF)

S13 Fig Episomal plasmids derived for the ectopic expression of epigenetically-defined biomarkers in mammalian cells. (TIF)

S14 Fig Epigenetically-regulated biomarkers, though required for pluripotency, do not necessarily have a role in reprogramming of fibroblasts to iPSCs. Transfection of human dermal fibroblasts (HDFs) with either a set of three episomal plasmids expressing OCT4, SOX2, KLF4, L-MYC, LIN28 and a short hairpin RNA directed against p53 (Y4) alone (left-most column) or with the Y4 set supplemented with either HMGA1, GLIS2 or PFDN5 (See S12 Fig), or all three (Y4 + HMGA1/GLIS2/PFDN5), generated phenotypically pluripotent colonies as assessed by alkaline phosphatase staining (No. AP+ Colonies). Three independent experiments are shown, (A, B), *n* = 10 independent transfections for each condition; (C) *n* = 5. Statistically significant differences in AP+ colony number (from Y4 alone) are indicated by asterisks: *, P≤0.05, **, P ≤0.01, ***, P ≤ 0.001, **** ≤ 0.0001.
(TIF)

S15 Fig Graphical summary showing the steps leading to identification of epigenetically-defined markers of pluripotency. (TIF)

S1 Table CGIs methylated in hESCs. (ZIP)

S2 Table Genes whose associated CGI is consistently methylated in hESCs. (ZIP)

S3 Table Human CGI array. Table giving location information and Affymetrix probe IDs of associated genes, if any. Location data refer to human genome build NCBI 36 (hg18).
(ZIP)

S4 Table The number of gene-associated CGIs on each chromosome. (ZIP)

S5 Table CGI methylation dataset for all of the hESC lines and somatic tissues. Correlated expression results, relative between the two lines indicated, are also included. U = CGI designated as unmethylated, M = CGI designated as methylated.
(ZIP)

S6 Table Transcriptome dataset for the female hESC lines RH1, RH3 and RCM1. Table showing RMA-normalised results for all probes for each individual sample, together with essential gene details and the mean probe signal for each line.
(ZIP)
