# Supplementary material for: Novel Human Embryonic Stem Cell Regulators Identified by Conserved and Distinct CpG Island Methylation State
Source: PLoS One. 2015 Jul 7;10(7):e0131102. doi: 10.1371/journal.pone.0131102 (PMC4495055; doi:10.1371/journal.pone.0131102)
Supplement: S1 Document — (A) X-inactivation status of female hESC lines RH1, RH3 and RCM1. (B) The relationship between expression profile and genomic methylation, and cell line derivation and culture conditions. (DOCX) [file pone.0131102.s001.docx]

**Novel human embryonic stem cell regulators identified by conserved and distinct CpG island methylation state: Supplementary Discussion**

Pells S.C., Koutsouraki, E., Morfopoulou S., Valencia-Cadavid, S., Tomlinson S.R., Kalathur, R., Futschik M.E., De Sousa P.A.

**A. X-Inactivation status of Female hESC Lines RH1, RH3 and RCM1.**

The hESC lines fell into two groups, with RH1 and RCM1 both having high rates of X-linked gene-associated CGI methylation (137 Me-GA-CGIs in RH1 and 138 in RCM1), and RH3 and RH4 having low rates of X-linked CGI methylation (39 Me-GA-CGIs in RH3, and 33 in the male line RH4, Table A in S1 Tables). Whilst the numbers of Me-GA-CGIs observed for RH3 and RH4 are expected, those for RH1 and RCM1 are strongly statistically significantly higher than expected (Table A in S1 Tables). These data are consistent with lines RH1 and RCM1 having undergone hypermethylation of the X chromosome as part of the X inactivation process, and RH3 not having done so. When considering differential CGI methylation (i.e., comparing any two lines with each other) on the X chromosome specifically, RH4 has only 2-4 genes methylated with respect to the female lines RH1 and RCM1, similar to RH3 (6-12). However, Both RCM1 and RH1 have > 100 genes methylated which are unmethylated in RH3 and RH4, but similar, lower (29/46 CGIs respectively) levels of X-linked CGI methylation when compared to each other. Thus whilst "overlap" between differential methylation and differential expression when comparing any two lines is not significant in most comparisons between any two lines included in our study (Table H in S1 Tables), there is usually strong signficance for the female hESC lines when considering X-linked genes specifically, where differential expression corresponds to differential methylation (i.e., methylated CGIs and underexpressed genes are taken together) (Table I in S1 Tables). These differential expression data are consistent with the Me-GA-CGI results for the X chromosome considered above. Similarly, although ANOVA confirmed very few statistically significant differences in expression of pseudoautosomal region (PAR)-located genes between any of RH1, RH3 and RCM1, as expected (S6 Fig.), examination of microarray expression data for RH1, RH3 and RCM1 showed expression of genes on the X chromosome is overall lower in RH1 and RCM1 (and similar to each other) compared to RH3, again, with the exception of genes at the telomeric end of the short arm of the X chromosome (PAR1, location Xp22.3) (S6 Fig.). However, only RCM1 expresses XIST, characteristic of female cells that have inactivated the X chromosome by coating of the inactive X chromosome with an XIST RNA "cloud" to generate XaXi cells expressing X-linked genes monoallelically (Table N in S1 Tables).

Female human ES cells differ from murine ESC lines derived from preimplantation blostocysts in that most have undergone X inactivation[[1-3](#_ENREF_1)]. Three classes of X chromosome status in female hESC lines are known. XIST expression is not necessary for maintenance of monoallelic expression of X linked genes[[4](#_ENREF_4)] and some hESC lines maintain XCI in the absence of XIST. This has been attributed to oxidative stress-mediated induction of XCI[[5](#_ENREF_5)],as hESCs derived in more physiological concentrations of oxygen (e.g. 5% O2) tend to remain XaXa, known as "Class I", whereas Class II hESCs have initiated XCI and maintain an XIST-coated inactive chromosome in the undifferentiated state. Class II cells may then switch to Class III and maintain monoallelic (XaXi) X chromosomal expression, even though they have lost XIST expression (reviewed in[[6](#_ENREF_6)]). Taken together, our data for the female hESC lines RH1, RH3 and RCM1 suggest that there is a representative of each class in this group of lines. RH3 is Class I (XaXa, with low levels of X chromosomal CGI methylation similar to that of the male line RH4, and to CGI methylation elsewhere in the genome). It does not express XIST (Table N in S1 Tables), and shows generally higher levels of X-linked expression throughout the X chromosome when compared to RH1 and RCM1 (S6 Fig.). RCM1 is Class II (XaXi, with high levels of X chromosomal CGI methylation and expression of XIST). RH1 is Class III, XaXi, with Xi maintenance via hypermethylation of the inactive X chromosome and probable associated repressive chromatin modifications but without XIST expression. Consistent with this interpretation, in the case of SCML1 (S3 Fig.), CGI methylation is on one allele and thus the gene is still expressed.

RH1 and RH3 are sibling embryos, both derived in normal atmospheric oxygen concentrations from surplus preimplantation blastocysts. They differed in their derivation method however, with RH3 being isolated in defined medium (XVivo-10 on laminin) in the absence of feeder fibroblasts, whereas RH1 was derived in standard conditions, in HDF-CM on Matrigel with feeder fibroblasts (Fletcher *et al*., 2006). RCM1 was derived in conditions similar to those of RH1, but from a failed-to-fertilise artificially activated oocyte (De Sousa, *et* al., 2009). It is possible that while RH1 followed the "normal" pathway of development during derivation (i.e., blastocyst ICM composed of XaXa cells undergoing XCI during derivation in high [O2] to produce XIST- XaXi ES cells), the derivation conditions of RH3 prevented this change. During derivation, RH3 was provided with feeder fibroblasts for a short period (7 days after initiation of embryonic cell attachment and outgrowth but was otherwise maintained in X-Vivo-10) on laminin in the absence of feeder cells.

**B. The Relationship between Expression Profile and Genomic Methylation, and Cell Line Derivation and Culture Conditions**

RH1 and RH3 had very similar global CGI methylation profiles apart from those of their X-chromosomes (S4 Fig.). RH1 and RCM1 were derived under the same culture conditions but differed in their provenance (“natural” development vs requiring an artificial activation stimulus to activate development (De Sousa et al., 2009; Fletcher et al., 2006)). These two lines differed more substantially in their CGI methylation profile (S4 Fig.) but clustered together with each other and apart from RH3 in their mRNA expression profile (S5 Fig.). While the limited number of cell lines studied precludes definitive conclusions, our interpretation of these results is that culture environment will have a more pronounced effect on gene expression profile than DNA methylation in hESC lines, whereas embryo developmental progression prior to hESC derivation is likely to have a pronounced effect on the latter. There are two distinct demethylation events in the preimplantation embryo, a rapid (6-8 hours) event following fertilisation which demethylates the paternal pronucleus, accompanied by hydoxymethylation[[7](#_ENREF_7), [8](#_ENREF_8)], followed by a slower demethylation event that demethylates the entire genome slowly over several cycles of cell division[[8-10](#_ENREF_8)]. Subsequently, de novo methylation occurs in the inner cell mass (ICM) at the blastocyst stage to reset the pluripotent cell epigenome prior to development of the organism. In this context an embryo which requires an activation stimulus to initiate development and/or is slow to develop will probably possess a different epigenome at the time of stem cell derivation even when embryos are morphologically equivalent, resulting in an ES cell line with an atypical DNA methylation pattern (S4 Fig.).

This may be summarised as:

“*With Human Embryonic Stem Cells, Transcriptome reflects Ontology, and Epigenome Reflects Ontogeny”*.

**Additional References Pertaining to Supplementary Discussion**

1. Hall LL, Byron M, Butler J et al. X-inactivation reveals epigenetic anomalies in most hESC but identifies sublines that initiate as expected. **Journal of cellular physiology***.* 2008;216:445-452.

2. Shen Y, Matsuno Y, Fouse SD et al. X-inactivation in female human embryonic stem cells is in a nonrandom pattern and prone to epigenetic alterations. **Proceedings of the National Academy of Sciences of the United States of America***.* 2008;105:4709-4714.

3. Silva SS, Rowntree RK, Mekhoubad S et al. X-chromosome inactivation and epigenetic fluidity in human embryonic stem cells. **Proceedings of the National Academy of Sciences of the United States of America***.* 2008;105:4820-4825.

4. Wutz A, Rasmussen TP, Jaenisch R. Chromosomal silencing and localization are mediated by different domains of Xist RNA. **Nature genetics***.* 2002;30:167-174.

5. Lengner CJ, Gimelbrant AA, Erwin JA et al. Derivation of pre-X inactivation human embryonic stem cells under physiological oxygen concentrations. **Cell***.* 2010;141:872-883.

6. Minkovsky A, Patel S, Plath K. Concise review: Pluripotency and the transcriptional inactivation of the female Mammalian X chromosome. **Stem cells (Dayton, Ohio)***.* 2012;30:48-54.

7. Oswald J, Engemann S, Lane N et al. Active demethylation of the paternal genome in the mouse zygote. **Curr Biol***.* 2000;10:475-478.

8. Santos F, Hendrich B, Reik W et al. Dynamic reprogramming of DNA methylation in the early mouse embryo. **Developmental biology***.* 2002;241:172-182.

9. Monk M, Boubelik M, Lehnert S. Temporal and regional changes in DNA methylation in the embryonic, extraembryonic and germ cell lineages during mouse embryo development. **Development (Cambridge, England)***.* 1987;99:371-382.

10. Rougier N, Bourc'his D, Gomes DM et al. Chromosome methylation patterns during mammalian preimplantation development. **Genes & development***.* 1998;12:2108-2113.
