# Supplementary material for: Novel Human Embryonic Stem Cell Regulators Identified by Conserved and Distinct CpG Island Methylation State
Source: PLoS One. 2015 Jul 7;10(7):e0131102. doi: 10.1371/journal.pone.0131102 (PMC4495055; doi:10.1371/journal.pone.0131102)
Supplement: S1 File — (A). Summary of hESC lines employed in this study. (B) Methylation of X-linked gene-associated CGIs in hESC Lines. (C) CGIs removed from consideration as methylated in all hESC lines because of inconsistent behaviour between multiple reporters. (D) hESC-specific gene associated methylation. (E) Correlation of hESC-specific CGI methylation status and transcriptome. (F) Expressed genes with an associated CGI which is always methylated in hESCs and always unmethylated in somatic tissues. (G) Expressed genes with an associated CGI which is always unmethylated in hESCs and always methylated in somatic tissues. (H) Assessment of differential CGI methylation and differential gene expression between female hES lines. (I) Differentially methylated and expressed genes are strongly associated with the X chromosome in female hESC lines. (J) Enrichment and depletion of functional categories in Gene Ontology of genes with an associated CGI in the human genome. (K) Summary of GO analysis indicating over-represented functions of epigenetically defined biomarkers of hESCs. (L) Microarray probe data for selected candidate epigenetic biomarkers GLIS2, HMGA1 and PFDN5 in female hESC lines. (M) Sequences of siRNAs employed in this study. (N) Microarray probe data for XIST expression in female hESC lines. (O) Primers used for RT-qPCR analysis. (P) Primer sequences for amplification of indicated gene promoter region after chromatin immunoprecipitation for OCT4 in hESCs. (Q) Table listing unmethylated CGIs in hESCs and somatic tissues. (DOCX) [file pone.0131102.s002.docx]

**Novel human embryonic stem cell regulators identified by conserved and distinct CpG island methylation state: Supplementary Tables**

Pells S.C., Koutsouraki, E., Morfopoulou S., Valencia-Cadavid, S., Tomlinson S.R., Kalathur, R., Futschik M.E., De Sousa P.A.

| **ESC Line^Ref^** | **Passage** | **Embryo Origin** | **Sex** | **Medium** | **Substrate** |
| --- | --- | --- | --- | --- | --- |
| **RH1^1^** | 76 | Cleavage stage | ♀ | HDF-CM +bFGF/SR/NS | Matrigel |
| **RH3^1^** | 57 | Cleavage stage | ♀ | DM +bFGF/NS | Laminin |
| **RH4^1^** | 36 | Cleavage stage | ♂ | HDF-CM +bFGF/SR/NS | Matrigel |
| **RCM1^2^** | 50 | Failed egg | ♀ | HDF-CM +bFGF/SR/NS | Matrigel |

**Table A. Summary of hESC lines employed in this study.** Cell lines were isolated from blastocyst stage embryos as previously reported in Fletcher *et al*., 2006 [1] or De Sousa *et al*., 2009 [2]. These originated from either cleavage stage embryos or artificially activated failed-to-fertilise eggs, respectively. hESC lines were initially derived on human dermal fibroblast (HDF) feeder cells for up to 10 passages in either HDF-conditioned medium (HDF-CM) or X-Vivo10 defined medium (DM), supplemented with (+) bFGF, serum replacement (SR), and/or nutrient supplements (Glutamine, non-essential amino acids, β-mercaptoethanol; NS) followed by expansion and maintenance off feeders on either Matrigel^TM^ or human laminin. At the designated passage genomic DNA or mRNA was analysed as described in the methods by hybridisation of replicate sample sets to human CGI and Affymetrix U133plus2 gene chip arrays, respectively.

| **Line** | **Sex** | **XL-Me-GA-CGI (Observed)** | **XL-Me-GA-CGI (Expected)** | **P (Χ^2^)** |
| --- | --- | --- | --- | --- |
| **RH1** | ♀ | 137 | 47 | 3.98 x 10^-46^ |
| **RH3** | ♀ | 39 | 48 | 0.174 |
| **RH4** | ♂ | 33 | 38 | 0.419 |
| **RCM1** | ♀ | 138 | 41 | 9.23 x 10^-60^ |

**Table B. Methylation of X-linked gene-associated CGIs in hESC Lines.** X-linked gene-associated CGI methylation (XL-Me--GA-CGI) in human hESC lines shows strongly significant hypermethylation in RH1 and RCM1, but the expected levels of CGI methylation in RH3 and the male line RH4.

| I1507 | I286 | I5766 | I6015 | I6941 | I8479 | I8294 | I9575 |
| --- | --- | --- | --- | --- | --- | --- | --- |
| I9177 | I10875 | I10877 | I11210 | I10980 | I11855 | I12126 | I13421 |
| I12233 | I12302 | I14032 | I14381 | I17593 | I16940 | I19049 | I18636 |
| I18639 | I20774 | I20917 | I22259 | I24343 | I24361 | I24934 | I24440 |

**Table C.** CGIs removed from consideration as methylated in all hESC lines because of inconsistent behaviour between multiple reporters.

.

| **(i) Genes with Me-CGI in hESCs** | | | | | |  | **(ii) Genes with UnMe-CGI in hESCs** | | |
| --- | --- | --- | --- | --- | --- | --- | --- | --- | --- |
| ABHD12 | CTNND2 | JMJD2C | NGEF | RAB3GAP1 | TET1 |  | AC004834.2 | GLIS2 | REXO4 |
| ABHD14A | CTSA | KCNK12 | NHLRC1 | RAD51L1 | TFCP2 |  | AC025181.8 | GMIP | RLTPR |
| AC004876.2 | CUGBP2 | KIAA0182 | NHP2L1 | RASGRP2 | TFG |  | AL023882.4 | GNAS | RNASEH2C |
| AC006299.1 | D4ST1 | KIAA0980 | NKAPL | RASSF4 | TMEM161B |  | AL031282.1 | GPRIN2 | RNF7 |
| AC007114.8 | DAAM1 | KLHL35 | NKPD1 | RBBP6 | TMEM56 |  | AL645924.13 | HMGA1 | RP11-364B14.3 |
| AC073624.28 | DAZAP1 | KRT10 | NR2E1 | RBBP7 | TMEM99 |  | ALDH1L2 | HOMER3 | RP4-697K14.7 |
| ADAM11 | DOCK9 | LAMP1 | NTAN1 | RND2 | TNR |  | ANGEL1 | HS6ST1 | RP5-1077B9.4 |
| ADCY1 | DPP10 | LCOR | NTNG2 | RP11-85G18.4 | TP53INP2 |  | ANKRD39 | HSH2D | RRAS |
| ADRB1 | DRD1IP | LHX3 | NUMA1 | RPP25 | TPP2 |  | ANKRD53 | HTRA4 | RYR2 |
| AKAP2 | DSCR3 | LINGO1 | NUP160 | RSHL2 | TRIM39 |  | AP003068.3 | IQCB1 | S1PR2 |
| AL162431.17 | EGFLAM | LTB4R | ODC1 | RUFY1 | TSLP |  | APOB | IRAK3 | SCRN1 |
| AL353732.14 | EIF2B4 | LTB4R2 | OLFM1 | SACM1L | TSPAN13 |  | ASAH3L | KANK4 | SHB |
| AP3B2 | FAM102B | LTBP2 | OSR1 | SDR42E1 | TSPYL5 |  | BAIAP2L2 | KIRREL3 | SLC22A3 |
| ARHGEF17 | FAM125B | MAD1L1 | PAX1 | SEPT5 | TTBK2 |  | BCAR1 | LENG1 | SLC25A42 |
| ARMC9 | FAM174B | MAFA | PCDH17 | SFRS2 | TXNDC5 |  | BMP4 | LITAF | SLC5A10 |
| ASXL3 | FANCC | MAP3K9 | PCDHGA11 | SH3GL1 | UBP1 |  | C10orf30 | LPAR2 | SNRPA1 |
| ATN1 | FGF18 | MAPKAPK3 | PCDHGA2 | SHB | VGLL4 |  | C16orf28 | MAP2K1 | SOX17 |
| ATP11B | FKTN | MBD5 | PCDHGA3 | SHOX2 | VSTM2B |  | C16orf59 | MCM2 | SRRM1 |
| AZIN1 | FOXI3 | MBTPS1 | PCDHGA6 | SIAH2 | WDR52 |  | C18orf1 | MIB2 | ST6GALNAC6 |
| BCL6 | FOXJ2 | ME2 | PCDHGA8 | SLC12A4 | WIPF3 |  | C1orf174 | MSX1 | SUDS3 |
| C10orf11 | FOXQ1 | METAP1 | PCDHGB7 | SLC13A3 | WIPI1 |  | C1orf32 | MTM1 | TAF6L |
| C11orf91 | GABRB2 | MFSD11 | PDE4A | SLC16A8 | WNK4 |  | C20orf55 | MUC4 | TBX3 |
| C14orf37 | GALNT1 | MGMT | PDIA6 | SLC25A37 | WNT3A |  | C7orf26 | NEFH | TEAD1 |
| C1orf128 | GALNT9 | MLH1 | PDS5B | SLC2A14 | WTIP |  | CADM4 | P2RX4 | TJP1 |
| C1orf14 | GHSR | MOGAT3 | PDXDC1 | SLC37A3 | ZBTB20 |  | CARHSP1 | PCDHA11 | TMC4 |
| C4orf16 | GOLGA8B | MRPL44 | PFDN5 | SLC6A9 | ZBTB34 |  | CCDC56 | PCDHA12 | TMCO4 |
| C5orf38 | GRAMD1C | MRPL47 | PHLPP | SLC8A3 | ZFYVE16 |  | CD79A | PCDHA2 | TMEM56 |
| CARS | GRHL3 | MSL2 | PI4KA | SLK | ZNF219 |  | CDCA4 | PCDHA4 | TOMM5 |
| CBLN4 | GRIN2D | MTERFD2 | PLIN | SNAP29 | ZNF688 |  | CDH1 | PCDHA6 | TSNARE1 |
| CCDC127 | GRWD1 | MTFR1 | PNPLA6 | SNORA42 | ZYX |  | CDK10 | PCDHA9 | TTTY14 |
| CCDC36 | HDAC4 | MTHFD2L | PPM1J | SNX1 |  |  | CNTD1 | PCDHAC1 | U6atac |
| CCDC9 | HIP1 | MYBL2 | PRICKLE1 | SNX13 |  |  | COL23A1 | PDE4A | WDR60 |
| CCL28 | HMGCR | N6AMT2 | PRKAG2 | SNX17 |  |  | EAF2 | PGAM5 | ZC3H12D |
| CCM2 | hsa-mir-9-3 | NARG1 | PSMD3 | SPTBN4 |  |  | FAM83G | PITRM1 | ZFPL1 |
| CIDEB | HUNK | NDUFB5 | PTGER3 | ST6GALNAC6 |  |  | FOXC1 | PLEKHA2 | ZMIZ1 |
| COX16 | ID4 | NDUFC1 | PTPRN2 | SUV39H2 |  |  | FRAT2 | PRODH | ZNF354B |
| CRI2 | IGFBP3 | NEURL2 | QRICH1 | SYCP1 |  |  | GAS2L1 | RAB40C | ZNF586 |
| CSMD3 | IL28RA | NFATC1 | RAB15 | TBX18 |  |  | GATA6 | RAI1 |  |

**Table D.** hESC-specific gene associated methylation. Genes whose associated CGI is (i) methylated in hESCs and unmethylated in somatic tissues or (ii) unmethylated in hESCs and methylated in somatic tissues.

| **hESC-Specific CGI Methylation State** | **mRNA Expression** | **No. Genes** |
| --- | --- | --- |
| **216 Methylated Genes**  **(201 CGIs)** | + | 128 |
|  | - | 76 |
|  | Differentially Exp^d^ (>1) | 12 |
| **109 Unmethylated Genes**  **(98 CGIs)** | + | 56 |
|  | - | 44 |
|  | Differentially Exp^d^ (>1) | 9 |

**Table E.** Correlation of hESC-specific CGI methylation status and transcriptome (Affymetrix U133plus2).

| ABHD12 | CTSA | GRIN2D | MAP3K9 | NARG1 | PNPLA6 | SH3GL1 | TMEM161B |
| --- | --- | --- | --- | --- | --- | --- | --- |
| SVIP | CUGBP2 | GRWD1 | MAPKAPK3 | NDUFB5 | PPM1J | SIAH2 | TMEM99 |
| ENGASE | CHST14 | HIP1 | MBD5 | NDUFC1 | PRICKLE1 | SLC12A4 | TP53INP2 |
| ARHGEF17 | DAAM1 | HMGCR | MBTPS1 | NFATC1 | PRKAG2 | SLC25A37 | TPP2 |
| ARMC9 | DAZAP1 | HUNK | ME2 | NHP2L1 | PSMD3 | SLC37A3 | TRIM39 |
| ATN1 | DOCK9 | ID4 | METAP1 | NTAN1 | QRICH1 | SLC8A3 | TSPAN13 |
| ATP11B | DSCR3 | IGFBP3 | MFSD11 | NUMA1 | RAB15 | SLK | TTBK2 |
| AZIN1 | EIF2B4 | IL28RA | MLH1 | NUP160 | RAB3GAP1 | SNAP29 | TXNDC5 |
| C1orf128 | FAM102B | JMJD2C | MRPL44 | ODC1 | RBBP6 | SNX1 | UBP1 |
| C4orf16 | FAM125B | KIAA0182 | MRPL47 | OLFM1 | RBBP7 | SNX13 | VGLL4 |
| CARS | FAM174B | NINL | MSL2 | PDIA6 | RND2 | SNX17 | WIPF3 |
| CCDC127 | FANCC | KRT10 | MTERFD2 | PDS5B | RPP25 | SPTBN4 | WIPI1 |
| CCDC9 | FKTN | LAMP1 | MTFR1 | PDXDC1 | RSPH3 | SUV39H2 | ZBTB34 |
| CCM2 | FOXJ2 | LCOR | MTHFD2L | PFDN5 | RUFY1 | TET1 | ZFYVE16 |
| COX16 | GALNT1 | LINGO1 | MYBL2 | PHLPP | SACM1L | TFCP2 | ZNF219 |
| EID2 | GOLGA8B | MAD1L1 | N6AMT2 | PI4KA | SFRS2 | TFG | ZYX |

**Table F.** Expressed genes with an associated CGI which is always methylated in hESCs and always unmethylated in somatic tissues.

| ANGEL1 | C1orf174 | EAF2 | HMGA1 | MCM2 | RAI1 | EDG5 | TEAD1 |
| --- | --- | --- | --- | --- | --- | --- | --- |
| ANKRD39 | C7orf26 | FAM83G | HOMER3 | P2RX4 | REXO4 | SCRN1 | TJP1 |
| ANKRD53 | CARHSP1 | FRAT2 | HS6ST1 | PGAM5 | RNASEH2C | SLC22A3 | TOMM5 |
| BCAR1 | CCDC56 | GAS2L1 | IQCB1 | PITRM1 | RNF7 | SLC25A42 | WDR60 |
| BMP4 | CDCA4 | GLIS2 | LITAF | PLEKHA2 | PRIC285 | SNRPA1 | ZFPL1 |
| C16orf28 | CDH1 | GNAS | EDG4 | PRODH | RP5-1077B9.4 | SRRM1 | ZMIZ1 |
| C16orf59 | CDK10 | GPRIN2 | MAP2K1 | RAB40C | RRAS | SUDS3 | ZNF586 |

**Table G.** Expressed genes with an associated CGI which is always unmethylated in hESCs and always methylated in somatic tissues

| **Methyln** | **Meth. Genes** | **Expressn** | **Expr. Genes** | **Me.-Exp. Overlap** | **P-value** |
| --- | --- | --- | --- | --- | --- |
| RH1 > RH3 | 562 | RH3 > RH1 | 656 | 80 | 3.58 x 10-16 |
| RH3 > RH1 | 506 | RH1 > RH3 | 690 | 28 | 0.59 |
| RH1 > RH3 | 562 | RH1 > RH3 | 690 | 34 | 0.36 |
| RH3 > RH1 | 506 | RH3 > RH1 | 656 | 25 | 0.83 |
|  | | | | | |
| RCM-1 > RH1 | 320 | RH1 > RCM1 | 345 | 29 | 4.38 x 10^-8^ |
| RH1 > RCM1 | 650 | RCM1 > RH1 | 299 | 23 | 0.049 |
| RCM1 > RH1 | 320 | RCM1 > RH1 | 299 | 4 | 0.96 |
| RH1 > RCM1 | 650 | RH1 > RCM1 | 345 | 21 | 0.29 |
|  | | | | | |
| RCM1 > RH3 | 505 | RH3 > RCM1 | 788 | 88 | 5.35 x 10^-18^ |
| RH3 > RCM1 | 752 | RCM1 > RH3 | 760 | 48 | 0.44 |
| RCM1 > RH3 | 505 | RCM1 > RH3 | 760 | 27 | 0.84 |
| RH3 > RCM1 | 752 | RH3 > RCM1 | 788 | 39 | 0.94 |

**Table H.** Assessment of differential CGI methylation and differential gene expression between female hES lines. *Line 1* > *Line 2* indicates genes whose associated CGI is methylated in line 1 and unmethylated in line 2 (column 1) or overexpressed in line compared to line 2 (column 3, defined as when log_2_[*Line 1 Exp/Line 2 Exp*] > 0.7 and *adjp*<0.1). "Meth. Genes" indicates the number of genes whose associated CGI is so differentially methylated between the two lines defined. Similarly, "Expr. Genes" indicates the number of so differentially expressed genes. "Me-Exp Overlap" indicates the number of genes for which both conditions are true, and P-value indicates the probability that this size of overlap is expected.

| **Meth^n^ / Expr^n^ Status** | ***N* (Genes)** | ***N* (Autosomal)** | ***N* (X-Linked)** | **Χ^2^** | **P** |
| --- | --- | --- | --- | --- | --- |
| **RH1^ME^ > RH3^ME^ / RH3^EXP^ > RH1^EXP^** | 80 | 30 | 50 | 1210.1 | 2.92x10^-265^ |
| **RCM1^ME^ > RH1^ME^ / RH1^EXP^ > RCM1^EXP^** | 29 | 17 | 12 | 184.4 | 5.38x10^-42^ |
| **RCM1^ME^ > RH3^ME^ / RH3^EXP^ > RCM1^EXP^** | 88 | 34 | 54 | 1281.2 | 1.38x10^-280^ |

**Table I.** Differentially Methylated and Expressed genes are strongly associated with the X chromosome in female hESC lines.

| **Enrichment** | **Depletion** |
| --- | --- |
| MF: Protein binding, DNA binding | MF: Receptors |
| MF: Transcription Factor Activity | MF: Signal Transducer |
| BP: Metabolism | BP: Immune System |
| BP: Cell Cycle | CC: Extracellular |
| BP: Embryonic Developmental Processes |  |
| CC: Intracellular Region |  |

**Table J**. Enrichment and depletion of functional categories in Gene Ontology of genes with an associated CGI in the human genome. (See also Figure S7).

|  | **GOMFID** | **P value** | **FDR** | **Odds Ratio** | **Exp Count** | **Count** | **No.** | **Term** |
| --- | --- | --- | --- | --- | --- | --- | --- | --- |
| **CGI-UnMe** | 0016563 | 0.00 | 0.01 | 6.48 | 1.64 | 9 | 247 | transcription activator activity |
|  | | | | | | | | |
| **CGI-Me** | 0016564 | 7.58 x 10^-4^ | 0.137166 | 3.697903 | 2.965029 | 10 | 197 | transcription repressor activity |

**Table K**. Summary of GO analysis indicating over-represented functions of epigenetically defined biomarkers of hESCs.

| **Probe** | **Gene** | **RH1** | **RH3** | **RCM1** |
| --- | --- | --- | --- | --- |
| 223378_at | GLIS2 | 7.89 | 7.41 | 8.06 |
|  | | | | |
| 210457_x_at | HMGA1 | 8.81 | 8.56 | 8.94 |
| 206074_s_at | HMGA1 | 12.38 | 12.30 | 12.20 |
|  | | | | |
| 207132_x_at | PFDN5 | 10.93 | 11.12 | 11.02 |
| 210908_s_at | PFDN5 | 10.93 | 11.11 | 11.05 |

**Table L.** Affymetrix U133Plus2 genechip probe data (Log2 probe signal for RMA-normalised data; three independent replicates for each cell line) for selected candidate epigenetic biomarkers GLIS2, HMGA1 and PFDN5 in female hESC lines. Expressed flags (50% genome) were declared when signal > 5.68 for RH1.

| **siRNA TARGET** | **SEQUENCE** |
| --- | --- |
| IDS-NULL (Negative Control) | GGCGACACCACCUAACAUU |
| YAP1 (Expressed Control) | UCACAUCGAUCAGACAACA |
| OCT4 (Positive Control) | AUCUUCAGGAGAUAUGCAA |
| GLIS2 | ACAAGCGCUAUUCCAACUC |
| HMGA1 | ACCACCACAACUCCAGGAA |
| PFDN5 | GGAUGCCAAGGACUUCUUC |

**Table M.** Sequences of siRNAs employed in this study. IDS-NULL targets no transcript produced by the human genome, and as such served as a negative control. YAP1 is expressed by hESCs but is dispensable with respect to maintenance of pluripotency, and as such provided a control to confirm specific knockdown and response in hESCs. OCT4 is a transcription factor required for pluripotency and its downregulation by siRNA has been shown to induce differentiation in both mouse and human ESCs.

| **Probe** | **RH1** | **RH3** | **RCM1** |
| --- | --- | --- | --- |
| 224590_at | 3.11 | 3.04 | 8.38 |
| 214218_s_at | 3.49 | 3.34 | 9.48 |
| 224589_at | 3.70 | 3.75 | 9.29 |
| 227671_at | 4.30 | 4.22 | 11.58 |
| 221728_x_at | 4.60 | 4.54 | 10.74 |
| 224588_at | 4.72 | 4.78 | 12.25 |
| 243712_at | 6.73 | 6.86 | 7.17 |

**Table N.** Affymetrix U133Plus2 genechip probe data (Log2 probe signal for RMA-normalised data; three independent replicates for each cell line) for *XIST* (Entrez reference 7503) expression in female hESC lines. Expressed flags (50% genome) were declared when signal > 5.68 for RH1.

| **Gene** | **Forward Primer** | **Reverse Primer** |
| --- | --- | --- |
| OCT4 | AGTGAGAGGCAACCTGGAGA | GCCTCAAAATCCTCTCGTTG |
| NANOG | AACTGGCCGAAGAATAGCAA | ACTGGATGTTCTGGGTCTGG |
| SOX2 | AGGGGGAAAGTAGTTTGCTGCCTCT | CTGCCGCCGCCGATGATTGT |
| GLIS2 | GCTCCCAGTCAGTGCTGTGTTGG | GGGTGGGGAAGTGCCTCTGAGAA |
| HMGA1 | AGGCATCCGCATTTGCTACCAGC | GAAGAGTGATGGCTGGGATGCGC |
| PFDN5 | AGAGCGTTGCTCGCCGAGAGA | GCTGAGCAATGGACGTGGACAAG |
| YAP1 | ACCGACCTGGAGGCGCTCTT | CTGGCCTGTCGGGAGTGGGA |
| GAPDH | GGCATGGACTGTGGTCATGAG | TGCACCACCAACTGCTTAGC |
| TET1 | GTAAATGGCCCCAAGTCAGA | CAGCTTCTGGGACATTAGCA |
| TET2 | GAGACGCTGAGGAAATACGG | TGGTGCCATAAGAGTGGACA |
| TET3 | CAGAACGCTGTGATCGTCAT | AACTTGCGAGGTGTCTTGCT |
| CDX2 | GAACCTGTGCGAGTGGATGCG | GGTCTATGGCTGTGGGTGGGAG |
| CGα | CCGCTCCTGATGTGCAGG | CTGTGACCCTGTTATATG |
| PL1 | ACACCTACCAGGAGTTTGAAG | AGTTTGTGTCAAACTTGC TGT |
| BRACHYURY | CCCGCGCACTACACACCCCTCACC | CCTTGGGCTGCGGCGTCGTACTG |
| BMP2 | CAGACCACCGGTTGGAGA | TCTCGGAAAACCTGAAGCTC |
| VEGF | AAACCTCACCAAGGCCAGC | CTTGCTCTATCTTTCTTTGGTCTGC |
| GATA2 | CCCTAAGCAGCGCAGCAAGAC | TGACTTCTCCTGCATGCACT |
| GATA4 | CATCAAGACGGAGCCTGGCC | TGACTGTCGGCCAAGACCAG |
| GATA6 | CCATGACTCCAACTTCCACC | ACGGAGGACGTGACTTCGGC |
| AFP | TCCCTCCTGCATTCTCTGAT | TCTGCAATGACAGCCTCAAG |
| ALBUMIN | GATGAGATGCCTGCTGACTTGC | CACGACAGAGTAATCAGGATGCC |
| HNF4a | AACACAATGCCCACTCACCT | CAGAGAGGGGCTTGACGAT |
| PAX6 | ACCAGCCCTTCGGTGAAT | TCACTTCCGGGAACTTGAAC |
| NESTIN | CAGCGTTGGAACAGAGGTTGG | TGGCACAGGTGTCTCAAGGGTAG |
| TUBULIN III | GGCCTTTGGACATCTCTTCA | GGCAGTCGCAGTTTTCACAC |
| NF-200 | CTTTCTCCCTTCCAGAAGGACT | ATCTCCTCTGTCTGTTCCTCCA |
| HAND1 | CCAAGGATGCACAGTCTGG | CGGTGCGTCCTTTAATCCT |

**Table O.** Primers used for RT-qPCR analysis.

| **Gene** | **Forward Primer** | **Reverse Primer** |
| --- | --- | --- |
| HMGA1 | GCCTTTCCTGCAGAGTCCTT | GAGGCCTGTCTTGGAGGAAC |
| GLIS2, R1 | CCCACAACGTCCTGAGCA | GCACAAGGCTTGGCACATAG |
| GLIS2, R2 | GCACAAGGCTTGGCACATAG | GCACAAGGCTTGGCACATAG |
| PFDN5 | TTCGTTAAGTCGGCCTTCCC | GGTTTCGCCTGGGAAAGGTA |

**Table P.** Primer sequences for amplification of indicated gene promoter region after chromatin immunoprecipitation for OCT4 in hESCs.

| **hESC Line** | **UnMe-CGI** | **% UnMe-CGI** | **UnMe-GA-CGI** | **% UnMe-GA-CGI** |
| --- | --- | --- | --- | --- |
| **RH1** | 14766 | 84.9 | 11663 | 79.0 |
| **RH3** | 14670 | 84.4 | 11594 | 79.0 |
| **RH4** | 15268 | 87.8 | 12022 | 78.7 |
| **RCM1** | 15076 | 86.7 | 11922 | 79.1 |
| **Adult Tissues** |  | | | |
| **Blood, ♂** | 15602 | 89.7 | 12323 | 79.0 |
| **Blood, ♀** | 15464 | 88.9 | 12192 | 79.1 |
| **Brain** | 15421 | 88.7 | 12190 | 79.0 |
| **Muscle** | 14841 | 85.4 | 11767 | 79.3 |
| **Spleen** | 14934 | 85.9 | 11817 | 79.1 |

**Table Q.** Unmethylated CGIs in human embryonic stem cell lines and adult somatic tissues. The number of unmethylated CGIs (UnMe-CGI) in each hESC line, and for adult somatic tissues (Illingworth *et al*., 2008), together with the percentage of CGIs that were unmethylated (% UnMe-CGI), and the number of unmethylated CGIs which are gene associated in these cell lines or tissues (UnMe-GA-CGI) and the proportion of unnmethylated CGIs that are gene-associated (% UnMe-GA-CGI).
