# Supplementary figures and images for: Novel Human Embryonic Stem Cell Regulators Identified by Conserved and Distinct CpG Island Methylation State
Source: PLoS One. 2015 Jul 7;10(7):e0131102. doi: 10.1371/journal.pone.0131102 (PMC4495055; doi:10.1371/journal.pone.0131102)

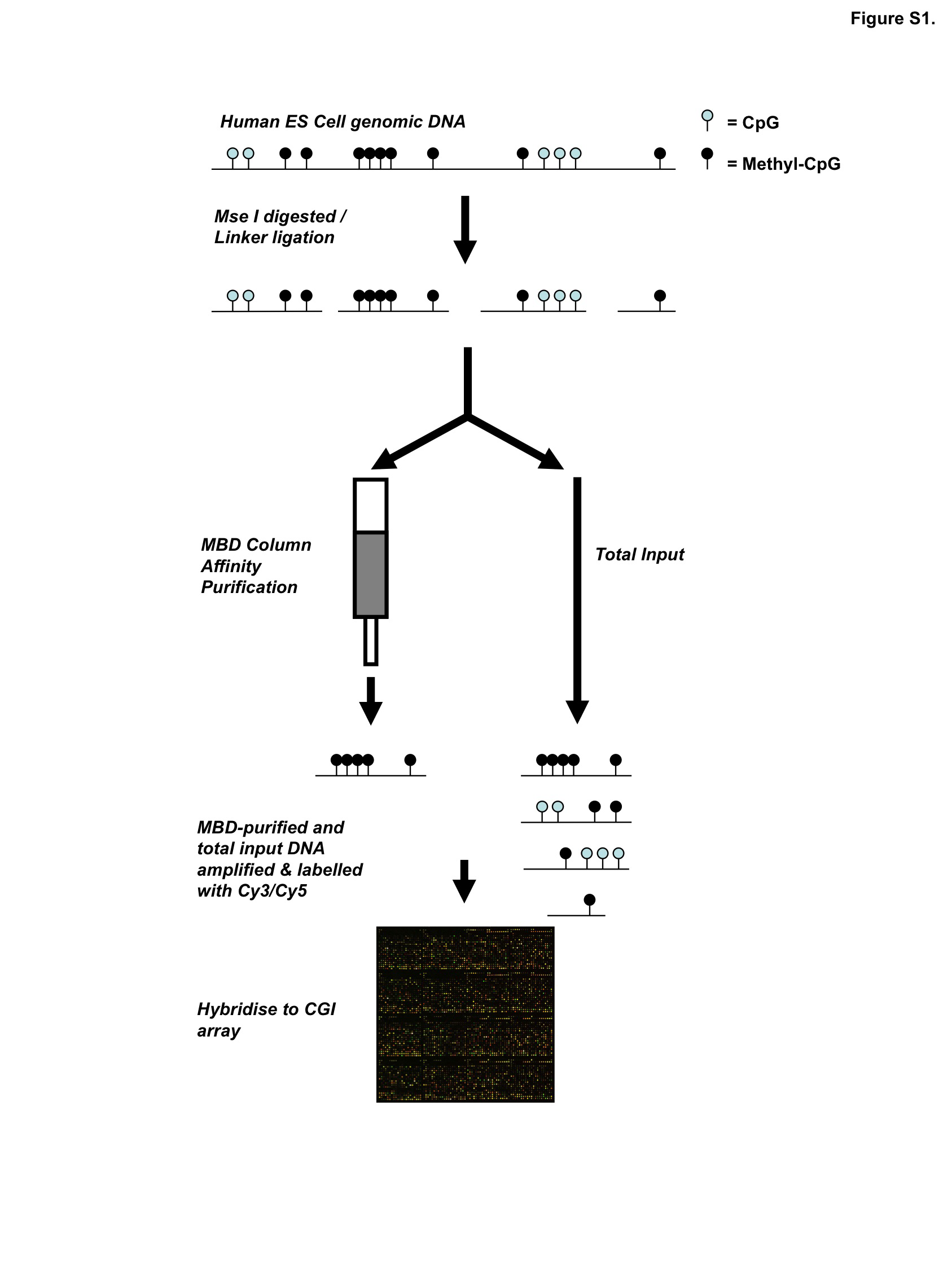

Supplement: S1 Fig — CGI Array Notes: CGI sequences on this array were identified on the basis of column-based binding of MseI-cloven genomic DNA from human blood mononuclear cells (pooled from 3 male donors) to a recombinant cysteine-rich CXXC3 domain of mouse Mbd1, which is characterised by a high affinity for non-methylated CpG sites (Voo et al., 2000; Jorgensen et al., 2004). MseI cleaves the sequence TTAA, and as such normal gDNA is cloven into small fragments (predicted average size 125 bp) containing typically 1 or 2 CpG dinucleotides. TTAA sites are underrepresented in CGIs, resulting in CGI-derived fragments of an average size of ~625 bp, and containing typically 50–60 unmethylated CpGs, enabling purification and subsequent sequencing of CGI-containing fragments. Because of Mbd1’s non-methylated CGI affinity based-purification, the resulting array excluded the small fraction of CGIs that are fully methylated in somatic cells, estimated to be less than 3% (Weber et al., 2007). Large scale sequencing of the column-bound fraction identified both CGIs predicted by CGI prediction algorithms and annotated in the ENSEMBL database, but also many CGIs that were predicted and only identified by their interaction with the mbd1 domain. As such, the CGIs on the array are biologically defined rather than defined by an algorithm. (TIF) [file pone.0131102.s003.tif]

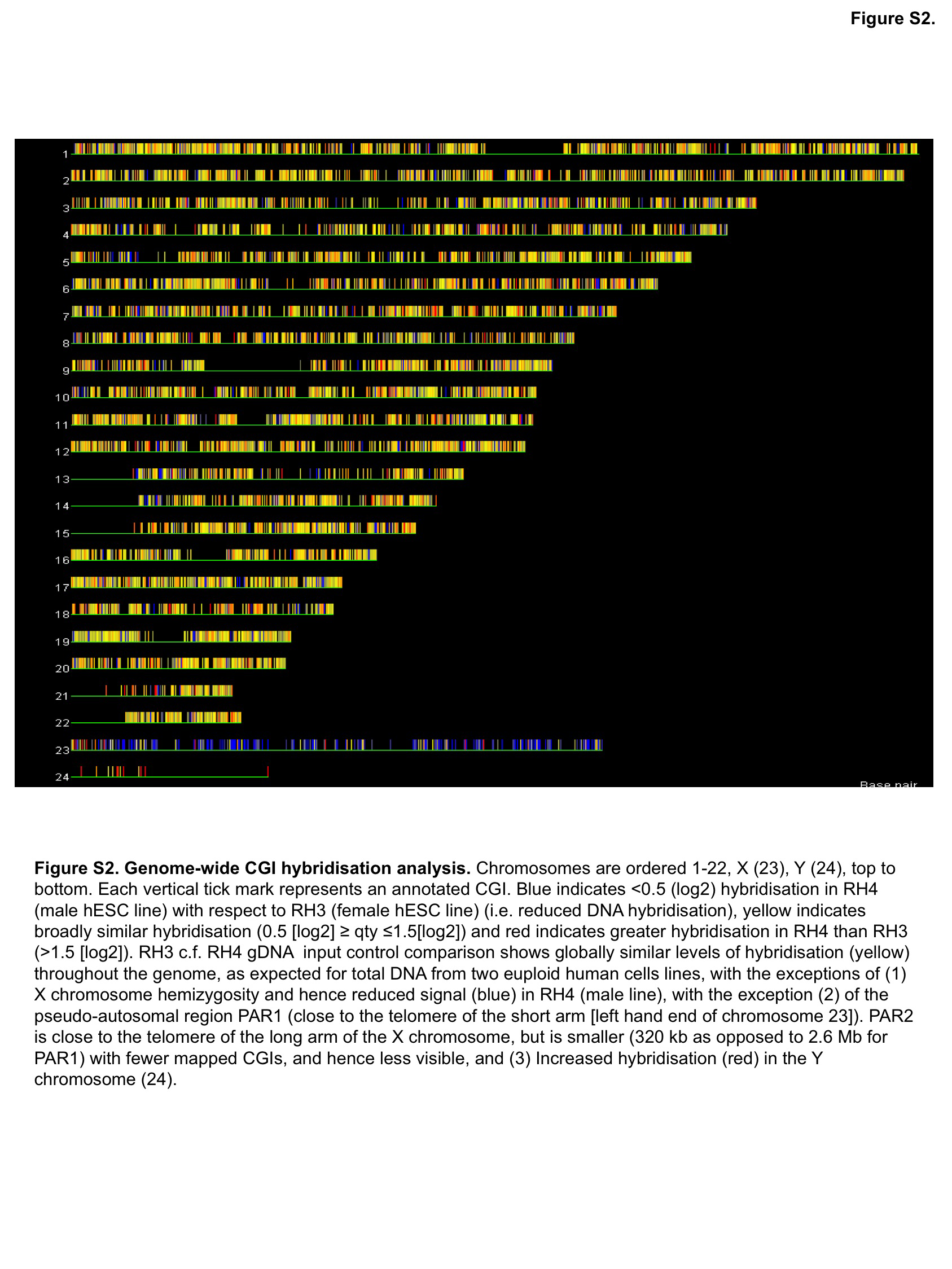

Supplement: S2 Fig — Chromosomes are ordered 1–22, X (23), Y (24), top to bottom. Each vertical tick mark represents an annotated CGI. Blue indicates <0.5 (log2) hybridisation in RH4 (male hESC line) with respect to RH3 (female hESC line) (i.e. reduced DNA hybridisation), yellow indicates broadly similar hybridisation (0.5 [log2] ≥ qty ≤1.5[log2]) and red indicates greater hybridisation in RH4 than RH3 (>1.5 [log2]). RH3 c.f. RH4 gDNA input control comparison shows globally similar levels of hybridisation (yellow) throughout the genome, as expected for total DNA from two euploid human cells lines, with the exceptions of (1) X chromosome hemizygosity and hence reduced signal (blue) in RH4 (male line), with the exception (2) of the pseudo-autosomal region PAR1 (close to the telomere of the short arm [left hand end of chromosome 23]). PAR2 is close to the telomere of the long arm of the X chromosome, but is smaller (320 kb as opposed to 2.6 Mb for PAR1) with fewer mapped CGIs, and hence less visible, and (3) Increased hybridisation (red) in the Y chromosome (24). (TIF) [file pone.0131102.s004.tif]

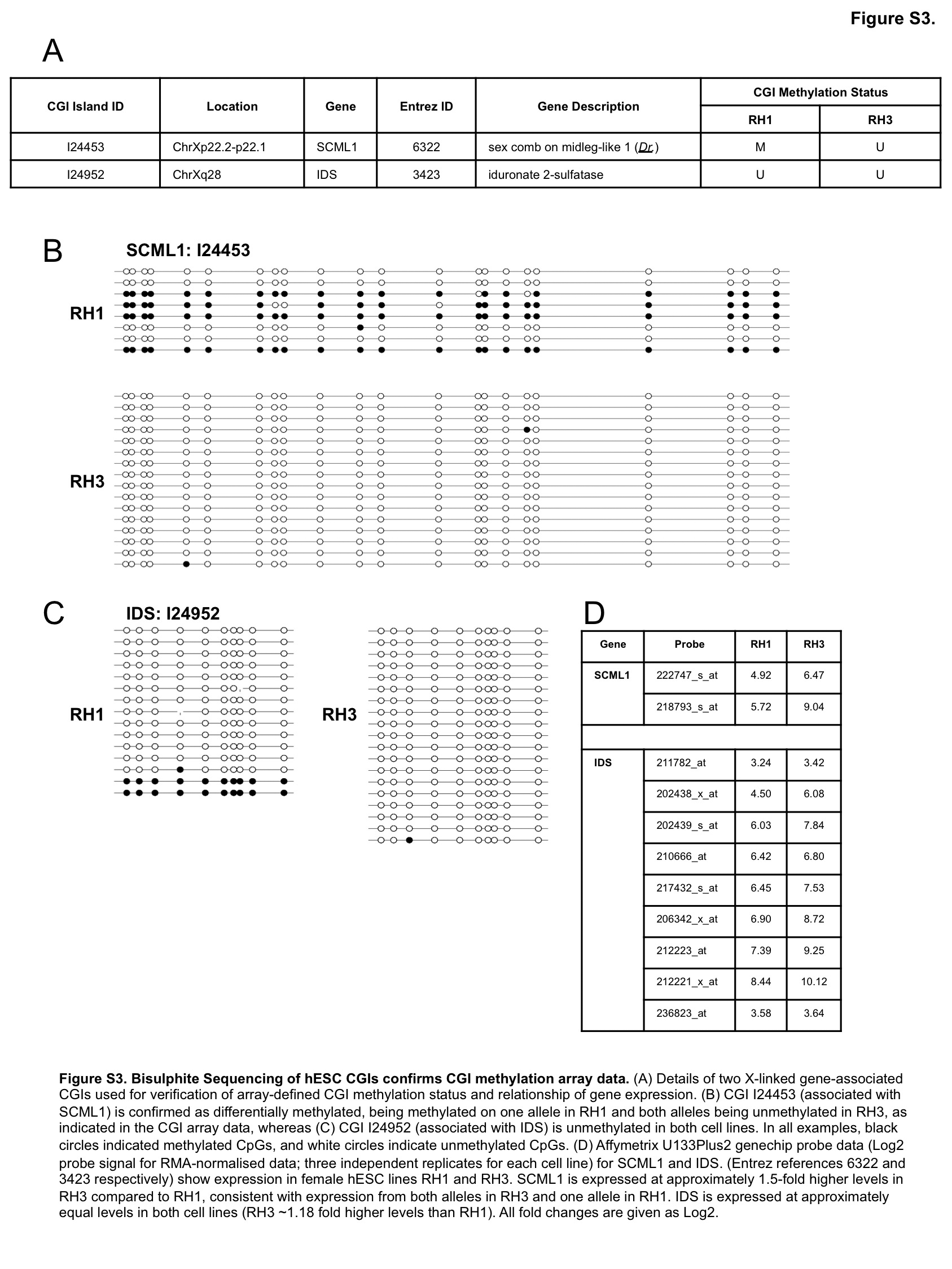

Supplement: S3 Fig — (A) Details of two X-linked gene-associated CGIs used for verification of array-defined CGI methylation status and relationship of gene expression. (B) CGI I24453 (associated with SCML1) is confirmed as differentially methylated, being methylated on one allele in RH1 and both alleles being unmethylated in RH3, as indicated in the CGI array data, whereas (C) CGI I24952 (associated with IDS) is unmethylated in both cell lines. In all examples, black circles indicated methylated CpGs, and white circles indicate unmethylated CpGs. (D) Affymetrix U133Plus2 genechip probe data (Log2 probe signal for RMA-normalised data; three independent replicates for each cell line) for SCML1 and IDS. (Entrez references 6322 and 3423 respectively) show expression in female hESC lines RH1 and RH3. SCML1 is expressed at approximately 1.5-fold higher levels in RH3 compared to RH1, consistent with expression from both alleles in RH3 and one allele in RH1. IDS is expressed at approximately equal levels in both cell lines (RH3 ~1.18 fold higher levels than RH1). All fold changes are given as Log2. (TIF) [file pone.0131102.s005.tif]

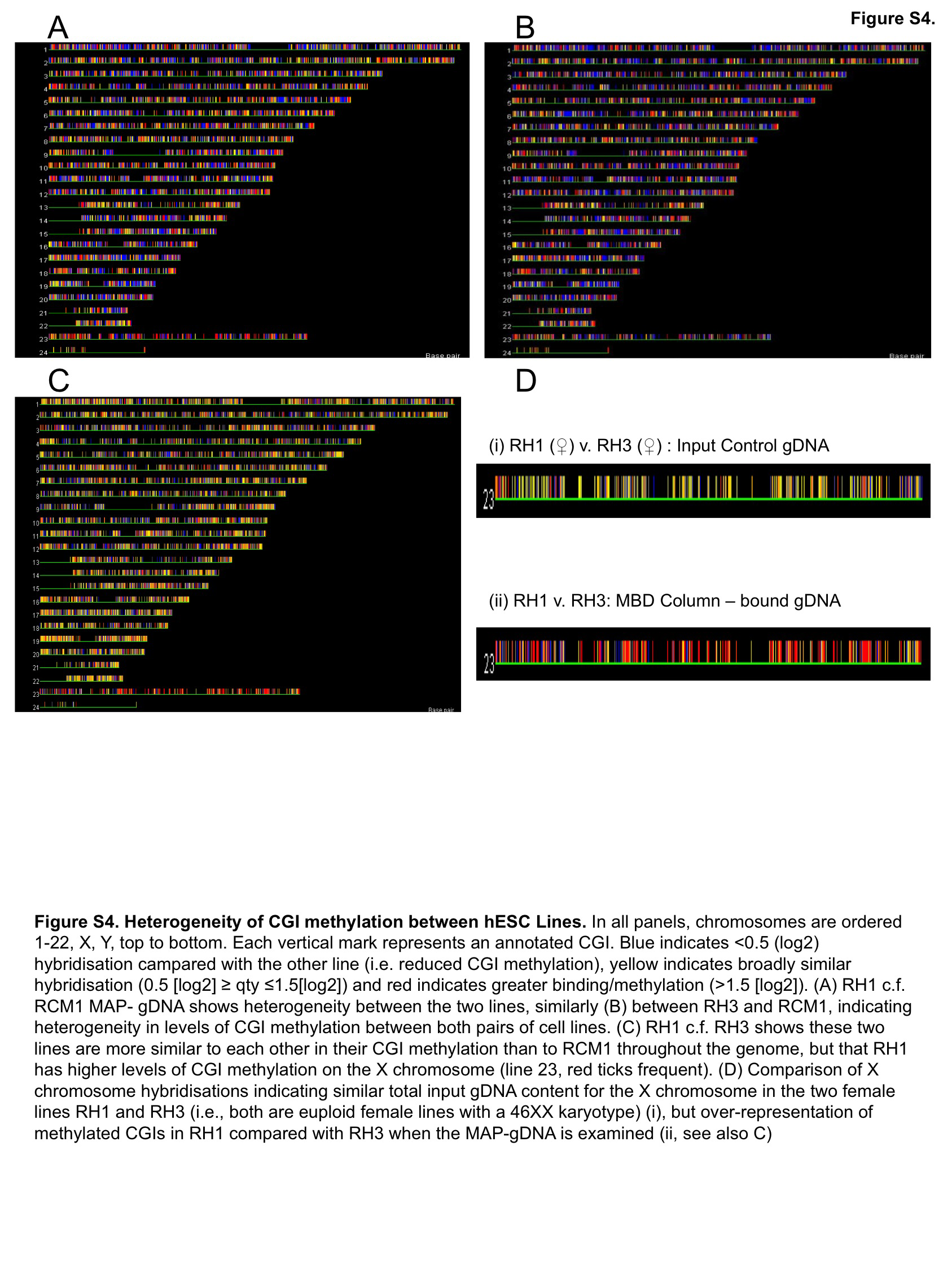

Supplement: S4 Fig — In all panels, chromosomes are ordered 1–22, X, Y, top to bottom. Each vertical mark represents an annotated CGI. Blue indicates <0.5 (log2) hybridisation compared with the other line (i.e. reduced CGI methylation), yellow indicates broadly similar hybridisation (0.5 [log2] ≥ qty ≤1.5[log2]) and red indicates greater binding/methylation (>1.5 [log2]). (A) RH1 c.f. RCM1 MAP- gDNA shows heterogeneity between the two lines, similarly (B) between RH3 and RCM1, indicating heterogeneity in levels of CGI methylation between both pairs of cell lines. (C) RH1 c.f. RH3 shows these two lines are more similar to each other in their CGI methylation than to RCM1 throughout the genome, but that RH1 has higher levels of CGI methylation on the X chromosome (line 23, red ticks frequent). (D) Comparison of X chromosome hybridisations indicating similar total input gDNA content for the X chromosome in the two female lines RH1 and RH3 (i.e., both are euploid female lines with a 46XX karyotype) (i), but over-representation of methylated CGIs in RH1 compared with RH3 when the MAP-gDNA is examined (ii, see also C) (TIF) [file pone.0131102.s006.tif]

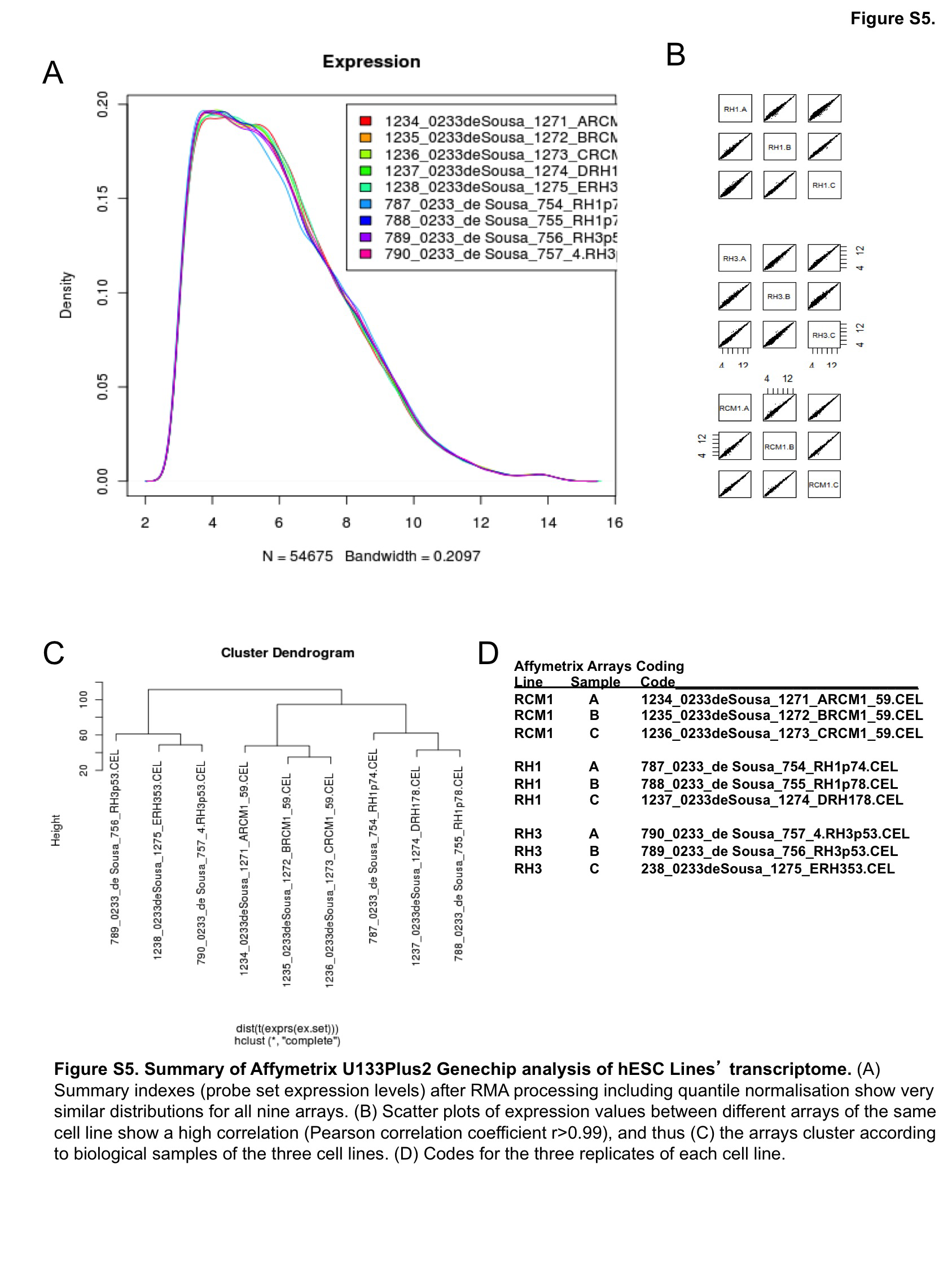

Supplement: S5 Fig — (A) Summary indexes (probe set expression levels) after RMA processing including quantile normalisation show very similar distributions for all nine arrays. (B) Scatter plots of expression values between different arrays of the same cell line show a high correlation (Pearson correlation coefficient r>0.99), and thus (C) the arrays cluster according to biological samples of the three cell lines. (D) Codes for the three replicates of each cell line. (TIF) [file pone.0131102.s007.tif]

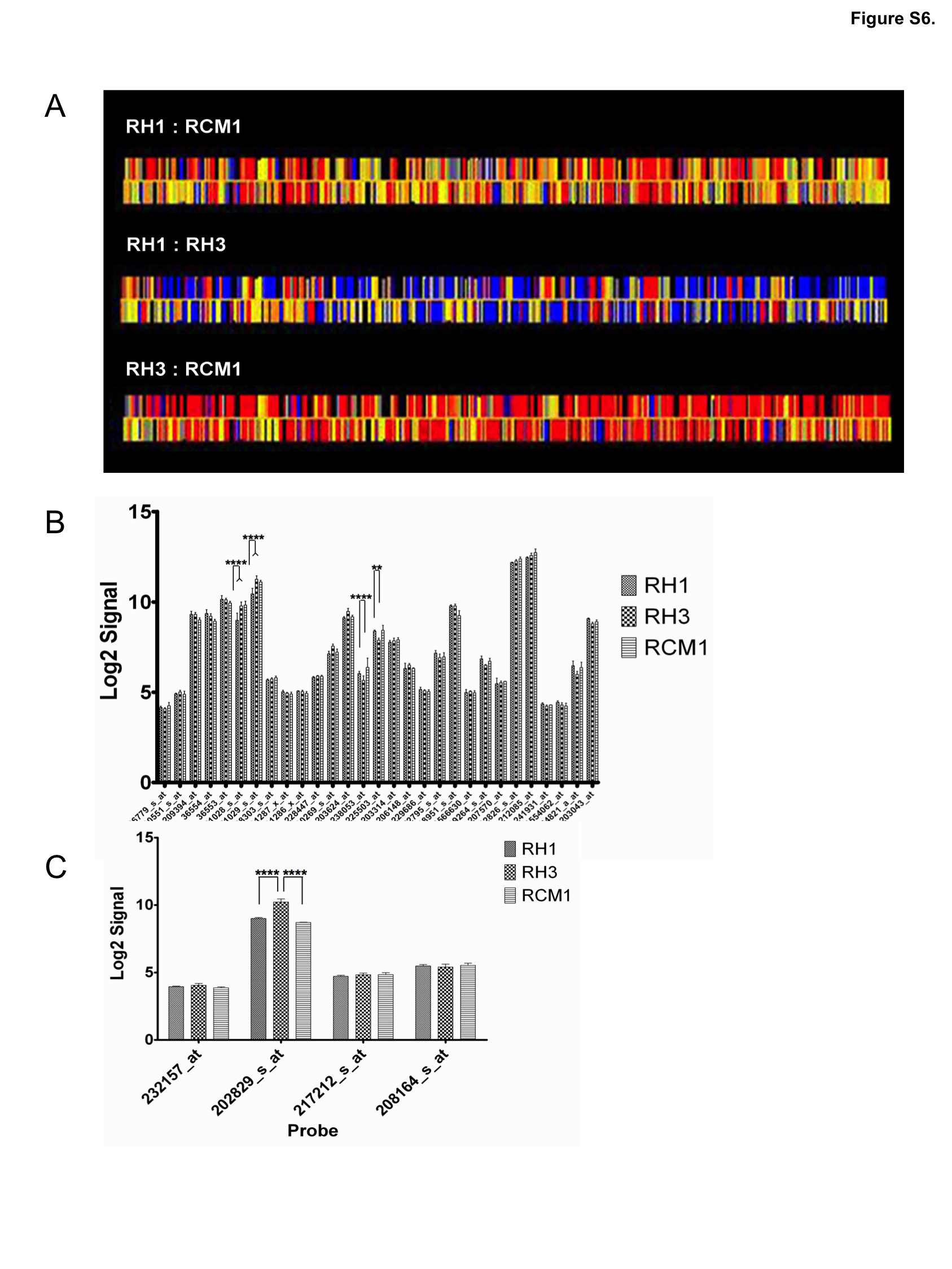

Supplement: S6 Fig — (A) Annotated X-linked probe relative expression level for RMA normalised data along the length of the X chromosome, comparing the pairs of cell lines indicated, shows broadly similar expression levels for RH1 and RCM1 (top panel; yellow tick indicates SignalRCM1 ≥ 0.75 x SignalRH1 and ≤ 1.5 x SignalRH1; red tick indicates SignalRH1 ≥ 1.5 x SignalRCM1 and blue tick indicates SignalRH1 < 0.75 x SignalRCM1). However, RH1 X-linked genes show frequently lower levels of expression when compared with RH3 (middle panel; blue ticks), with the exception of the pseudoautosomal region 1 located at Xp22 (left hand end, yellow ticks). Similarly, when RH3 is compared with RCM1 (lower panel), X-linked gene expression in RH3 is typically > 1.5-fold the levels observed in RCM1 (red ticks). Few significant biologically-relevant differences in expression of genes in the pseudoautosomal regions (B) PAR1 or (C) PAR2 were observed between the female hESC lines RH1, RH3, and RCM1. N = 3 independent replicates for each probe, plots indicate Log2 signal ± SD. (TIF) [file pone.0131102.s008.tif]

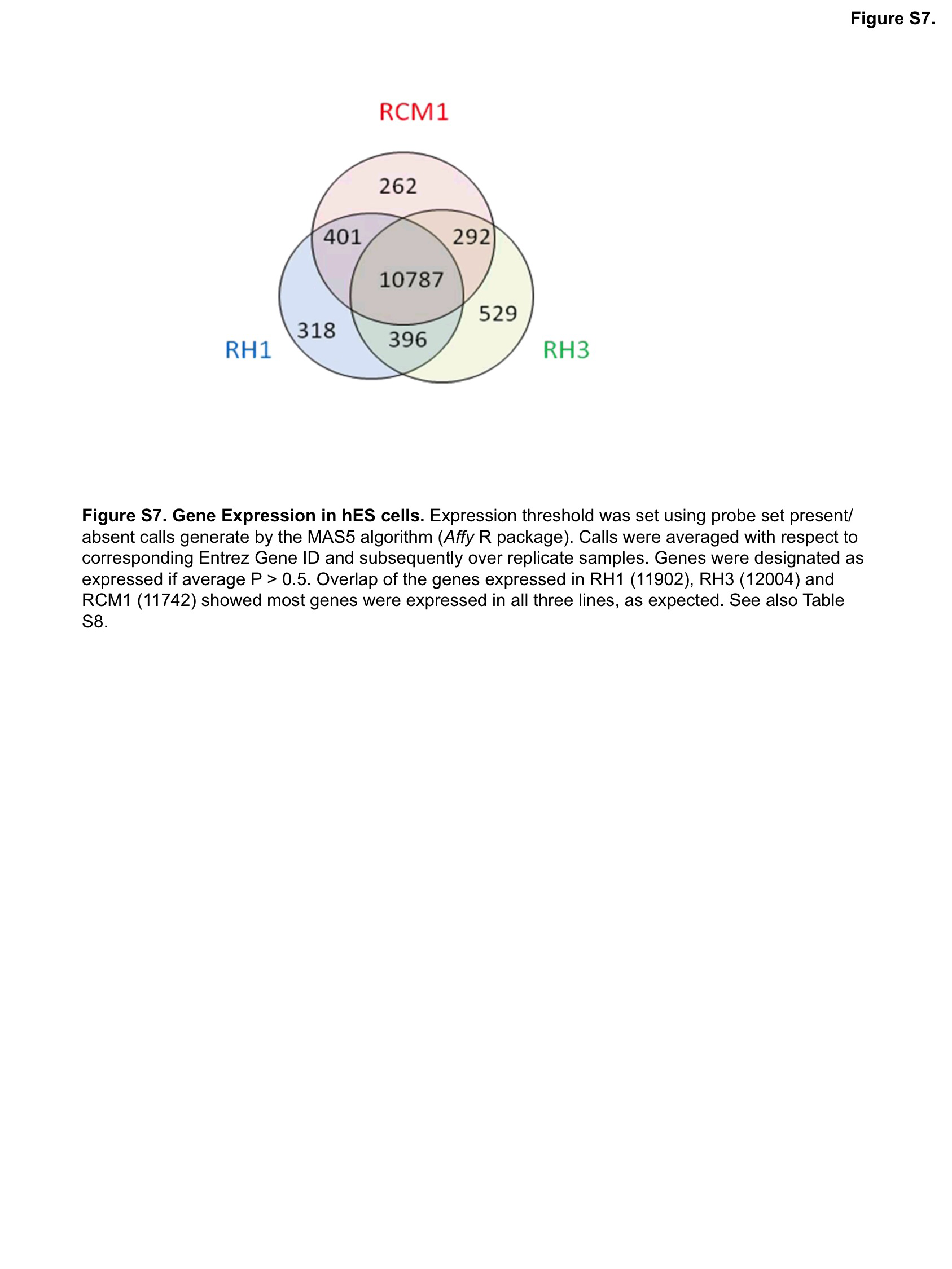

Supplement: S7 Fig — Expression threshold was set using probe set present/absent calls generate by the MAS5 algorithm (Affy R package). Calls were averaged with respect to corresponding Entrez Gene ID and subsequently over replicate samples. Genes were designated as expressed if average P > 0.5. Overlap of the genes expressed in RH1 (11902), RH3 (12004) and RCM1 (11742) showed most genes were expressed in all three lines, as expected. See also Table F in S1 File. (TIF) [file pone.0131102.s009.tif]

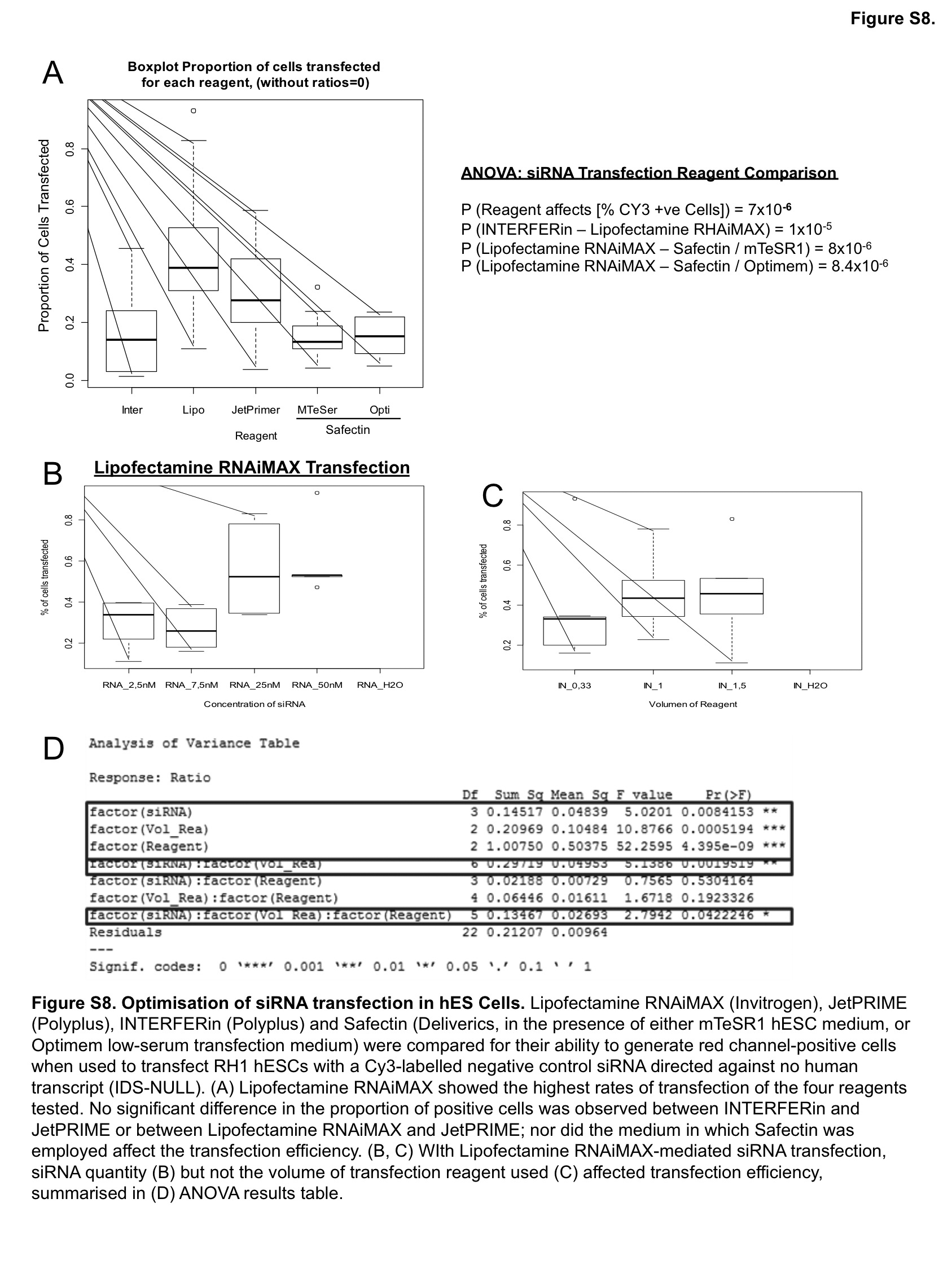

Supplement: S8 Fig — Lipofectamine RNAiMAX (Invitrogen), JetPRIME (Polyplus), INTERFERin (Polyplus) and Safectin (Deliverics, in the presence of either mTeSR1 hESC medium, or Optimem low-serum transfection medium) were compared for their ability to generate red channel-positive cells when used to transfect RH1 hESCs with a Cy3-labelled negative control siRNA directed against no human transcript (IDS-NULL). (A) Lipofectamine RNAiMAX showed the highest rates of transfection of the four reagents tested. No significant difference in the proportion of positive cells was observed between INTERFERin and JetPRIME or between Lipofectamine RNAiMAX and JetPRIME; nor did the medium in which Safectin was employed affect the transfection efficiency. (B, C) With Lipofectamine RNAiMAX-mediated siRNA transfection, siRNA quantity (B) but not the volume of transfection reagent used (C) affected transfection efficiency, summarised in (D) ANOVA results table. (TIF) [file pone.0131102.s010.tif]

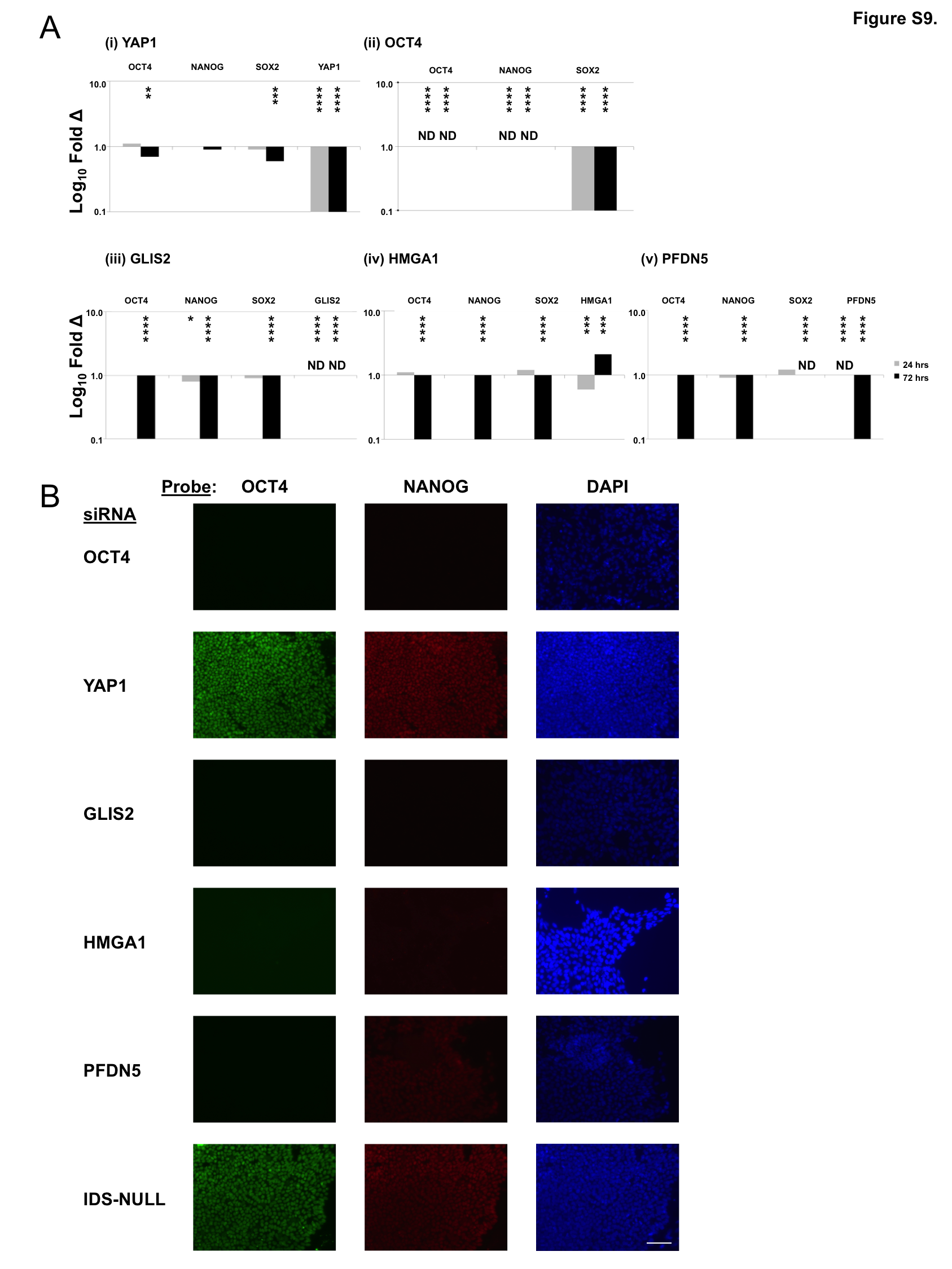

Supplement: S9 Fig — H9 hESCs were treated with siRNAs directed against the mRNAs indicated, with two treatments of siRNA at 0 and 24 hours. Samples were taken at 48 and 96 hours for gene expression analysis (24 and 72 hours after the final siRNA treatment). (A) RT-qPCR data showing log10 fold change in expression of the targeted gene, and associated effects on the stem cell transcription factors OCT4, NANOG and SOX2. Changes are relative to GAPDH expression, normalised to H9 hESCs treated with an siRNA which does not target any gene in the human genome (IDS-NULL). Asterisks indicate levels of statistical significance, as calculated by unpaired t-test (*≤0.05, **≤0.01, ***≤0.001, ****≤0.0001). ND: Not detected by 40 cycles of PCR. (i) Knockdown of YAP1, a gene expressed in hESCs but not required for a pluripotent phenotype was successful, but as expected had a minimal effect on the pluripotency markers OCT4, NANOG and SOX2. (ii) Knockdown of OCT4 also results in significant downregulation of associated pluripotency transcripts (NANOG and SOX2). (iii-v) In all cases where transcript levels of an epigenetically-defined biomarker was knocked down, (∞ indicates "infinite KD", i.e., no target transcript was detectable), the knockdown was accompanied by significant reductions in levels of the hESC markers OCT4, NANOG and SOX2. (B) Immunohistochemistry for the hESC markers NANOG and OCT4 at 72 hours after treatment with siRNAs directed against epigenetically defined biomarkers showed that protein levels of the pluripotency markers OCT4 and NANOG are downregulated in association with the reduction in mRNA transcript levels of GLIS2, HMGA1 or PFDN5. Scale bar = 100 μm. (TIF) [file pone.0131102.s011.tif]

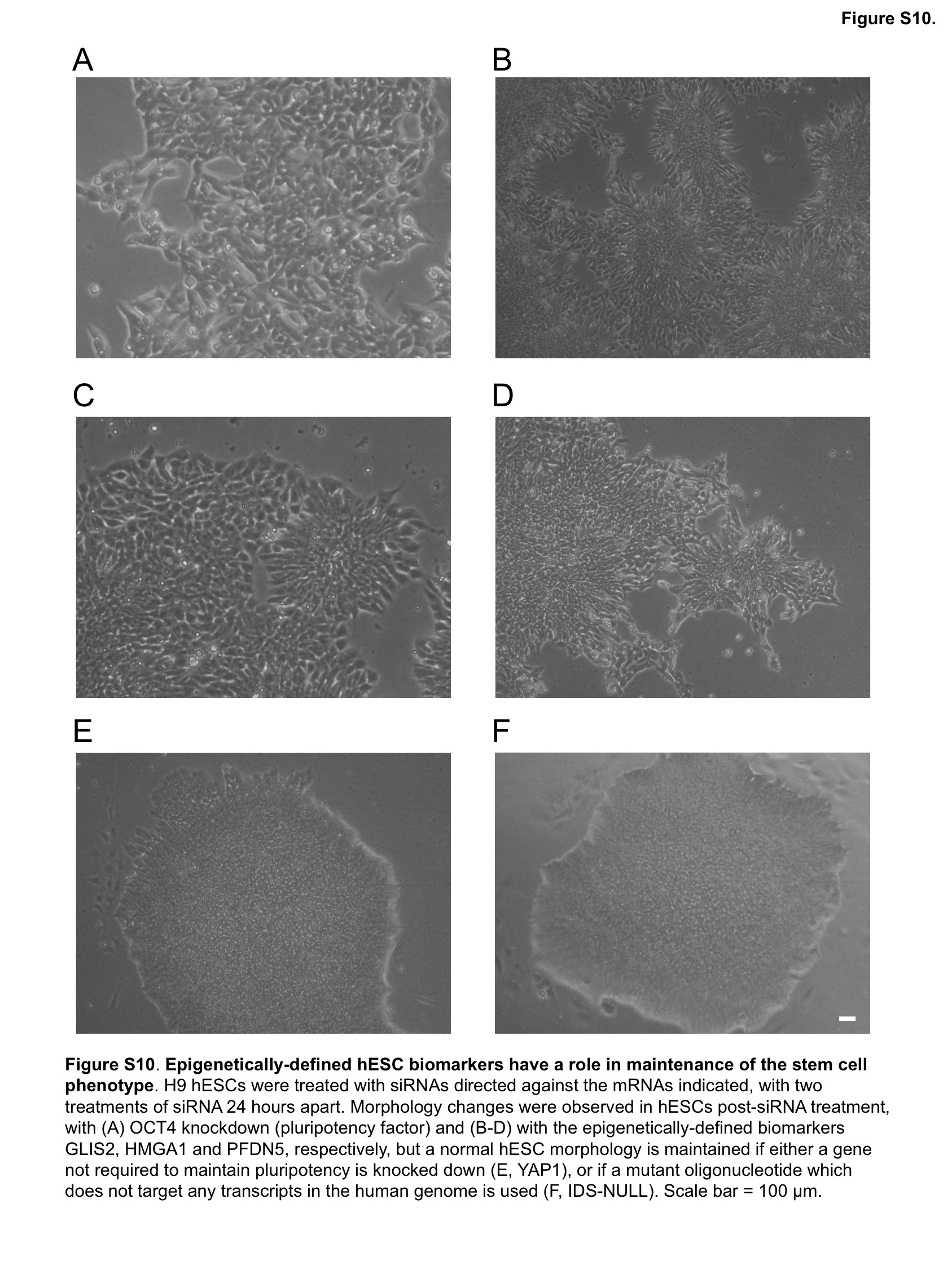

Supplement: S10 Fig — H9 hESCs were treated with siRNAs directed against the mRNAs indicated, with two treatments of siRNA 24 hours apart. Morphology changes were observed in hESCs post-siRNA treatment, with (A) OCT4 knockdown (pluripotency factor) and (B-D) with the epigenetically-defined biomarkers GLIS2, HMGA1 and PFDN5, respectively, but a normal hESC morphology is maintained if either a gene not required to maintain pluripotency is knocked down (E, YAP1), or if a mutant oligonucleotide which does not target any transcripts in the human genome is used (F, IDS-NULL). Scale bar = 100 μm. (TIF) [file pone.0131102.s012.tif]

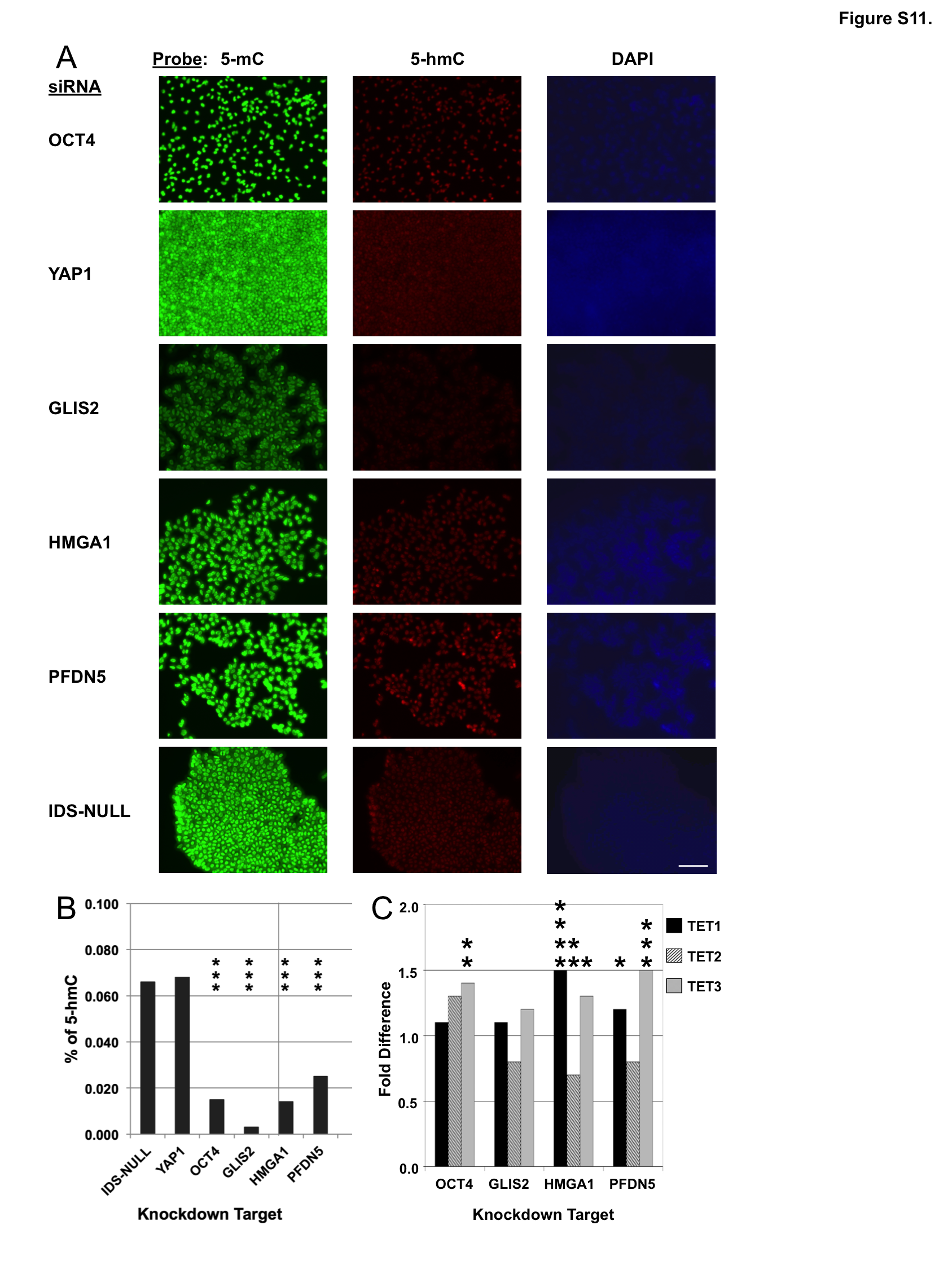

Supplement: S11 Fig — H9 hESCs were treated with siRNAs directed against the mRNAs indicated, with two treatments of siRNA 24 hours apart as described previously. (A) Immunohistochemical staining for the epigenetic marks 5-methylcytosine (5-mC) and 5-hydroxymethylcytosine (5-hmC) showed that after knockdown of OCT4, GLIS2, HMGA1 or PFDN5, the stem cell-associated mark 5-hmC becomes more difficult to detect. However, knockdown of either YAP1 or a negative control oligonucleotide (IDS-NULL) had no effect on 5-hmC in hESCs. Scale bar = 100 μm. (B) Quantification of 5-hmC levels by ELISA in cells as a percentage of total cytosine residues in genomic DNA confirms the result of immunochemical staining of 5-hmC, showing a large (>80%), statistically significant reduction in 5-hmC levels in H9 hESCs in which either OCT4 or one of the three epigenetically-defined biomarkers expression is knocked down by siRNA. (C) RT-qPCR data showing fold change in expression of the three TET oxidases in response to knockdown of OCT4 and the epigenetically defined biomarkers GLIS2, HMGA1 and PFDN5. Changes in TET enzyme transcript levels were mostly not significant or modest (within ~1.5-fold) in response to knockdown by OCT4, GLIS2, HMGA1 and PFDN5. Asterisks from 1–4 indicate levels of statistical significance calculated by ANOVA with Dunnett’s post-hoc test, in comparison to the IDS-NULL control. (TIF) [file pone.0131102.s013.tif]

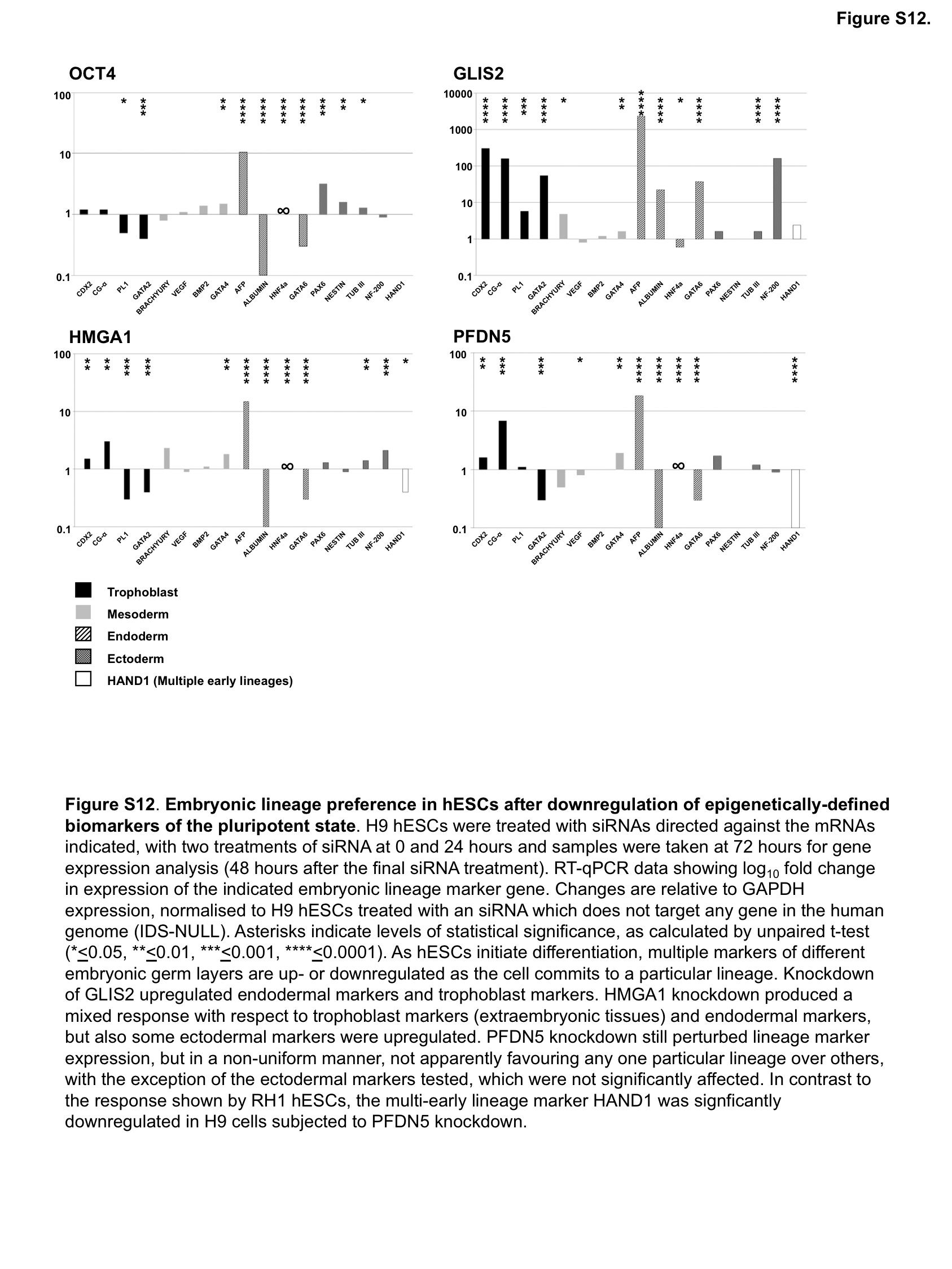

Supplement: S12 Fig — H9 hESCs were treated with siRNAs directed against the mRNAs indicated, with two treatments of siRNA at 0 and 24 hours and samples were taken at 72 hours for gene expression analysis (48 hours after the final siRNA treatment). RT-qPCR data showing log10 fold change in expression of the indicated embryonic lineage marker gene. Changes are relative to GAPDH expression, normalised to H9 hESCs treated with an siRNA which does not target any gene in the human genome (IDS-NULL). Asterisks indicate levels of statistical significance, as calculated by unpaired t-test (*0.05, **≤0.01, ***≤0.001, ****≤0.0001). ND: Not detected by 40 cycles of PCR. As hESCs initiate differentiation, multiple markers of different embryonic germ layers are up- or downregulated as the cell commits to a particular lineage. Knockdown of GLIS2 upregulated endodermal markers and trophoblast markers. HMGA1 knockdown produced a mixed response with respect to trophoblast markers (extraembryonic tissues) and endodermal markers, but also some ectodermal markers were upregulated. PFDN5 knockdown still perturbed lineage marker expression, but in a non-uniform manner, not apparently favouring any one particular lineage over others, with the exception of the ectodermal markers tested, which were not significantly affected. In contrast to the response shown by RH1 hESCs, the multi-early lineage marker HAND1 was significantly downregulated in H9 cells subjected to PFDN5 knockdown. (TIF) [file pone.0131102.s014.tif]

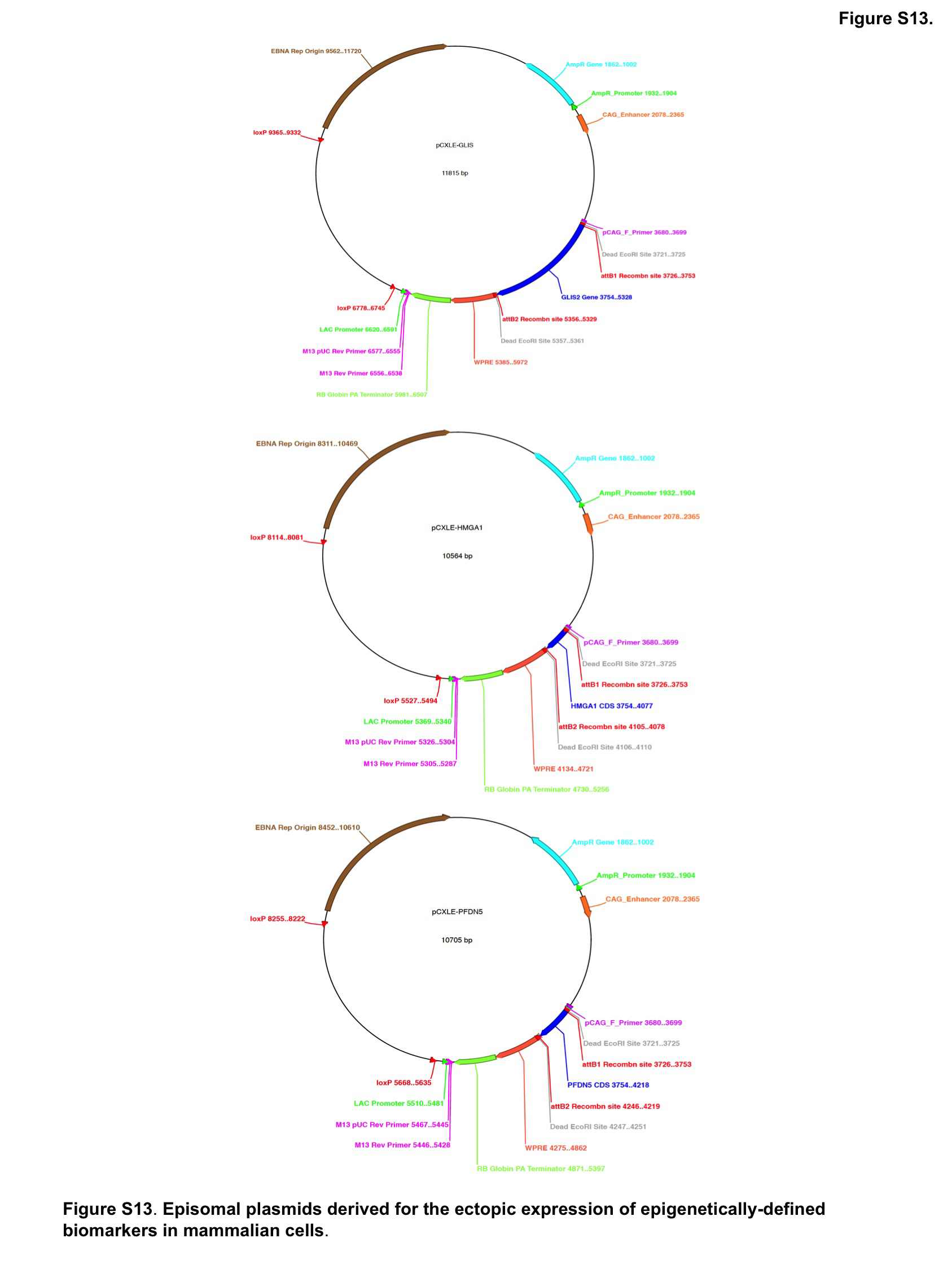

Supplement: S13 Fig — (TIF) [file pone.0131102.s015.tif]

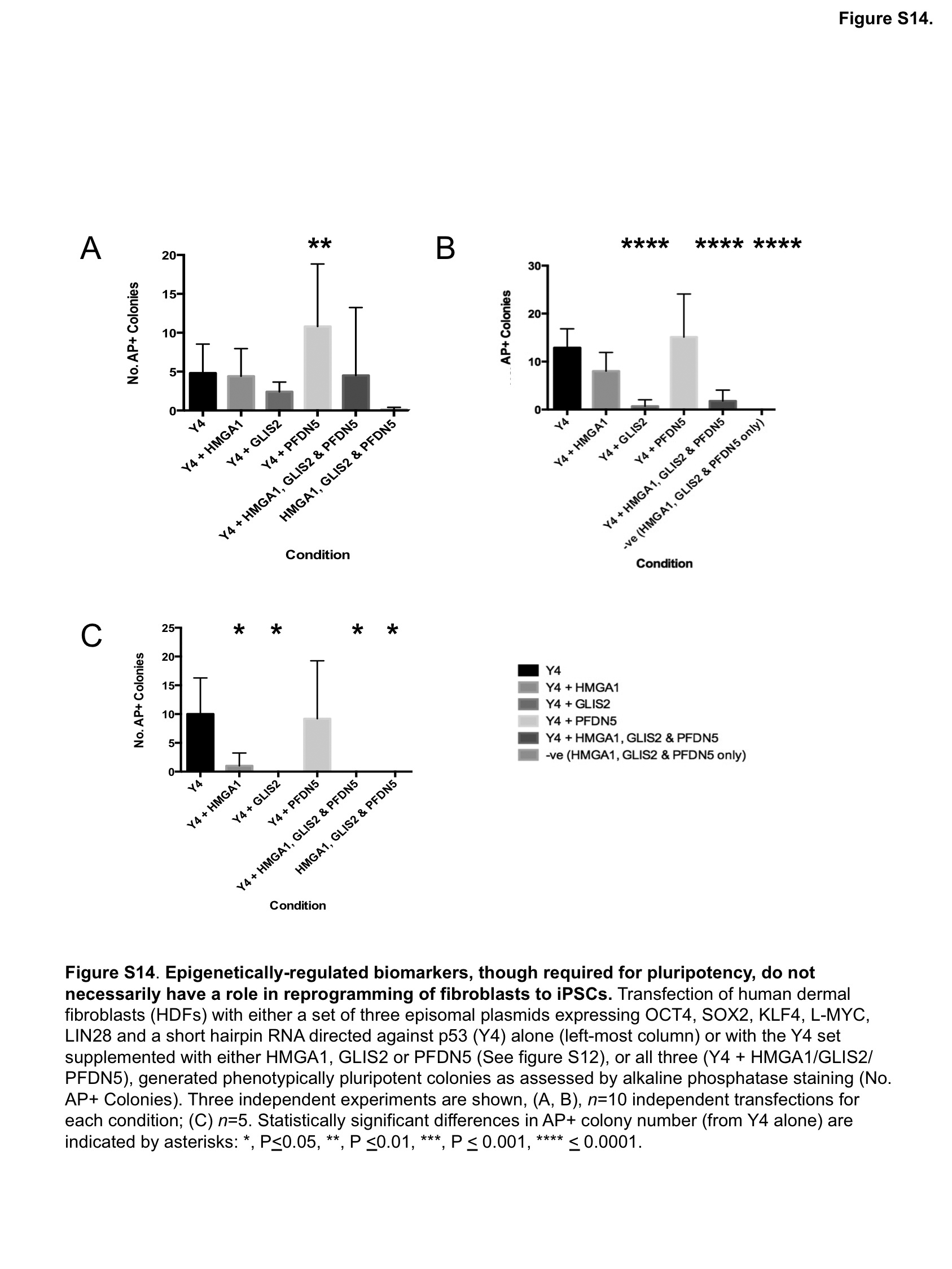

Supplement: S14 Fig — Transfection of human dermal fibroblasts (HDFs) with either a set of three episomal plasmids expressing OCT4, SOX2, KLF4, L-MYC, LIN28 and a short hairpin RNA directed against p53 (Y4) alone (left-most column) or with the Y4 set supplemented with either HMGA1, GLIS2 or PFDN5 (See S12 Fig), or all three (Y4 + HMGA1/GLIS2/PFDN5), generated phenotypically pluripotent colonies as assessed by alkaline phosphatase staining (No. AP+ Colonies). Three independent experiments are shown, (A, B), n = 10 independent transfections for each condition; (C) n = 5. Statistically significant differences in AP+ colony number (from Y4 alone) are indicated by asterisks: *, P≤0.05, **, P ≤0.01, ***, P ≤ 0.001, **** ≤ 0.0001. (TIF) [file pone.0131102.s016.tif]

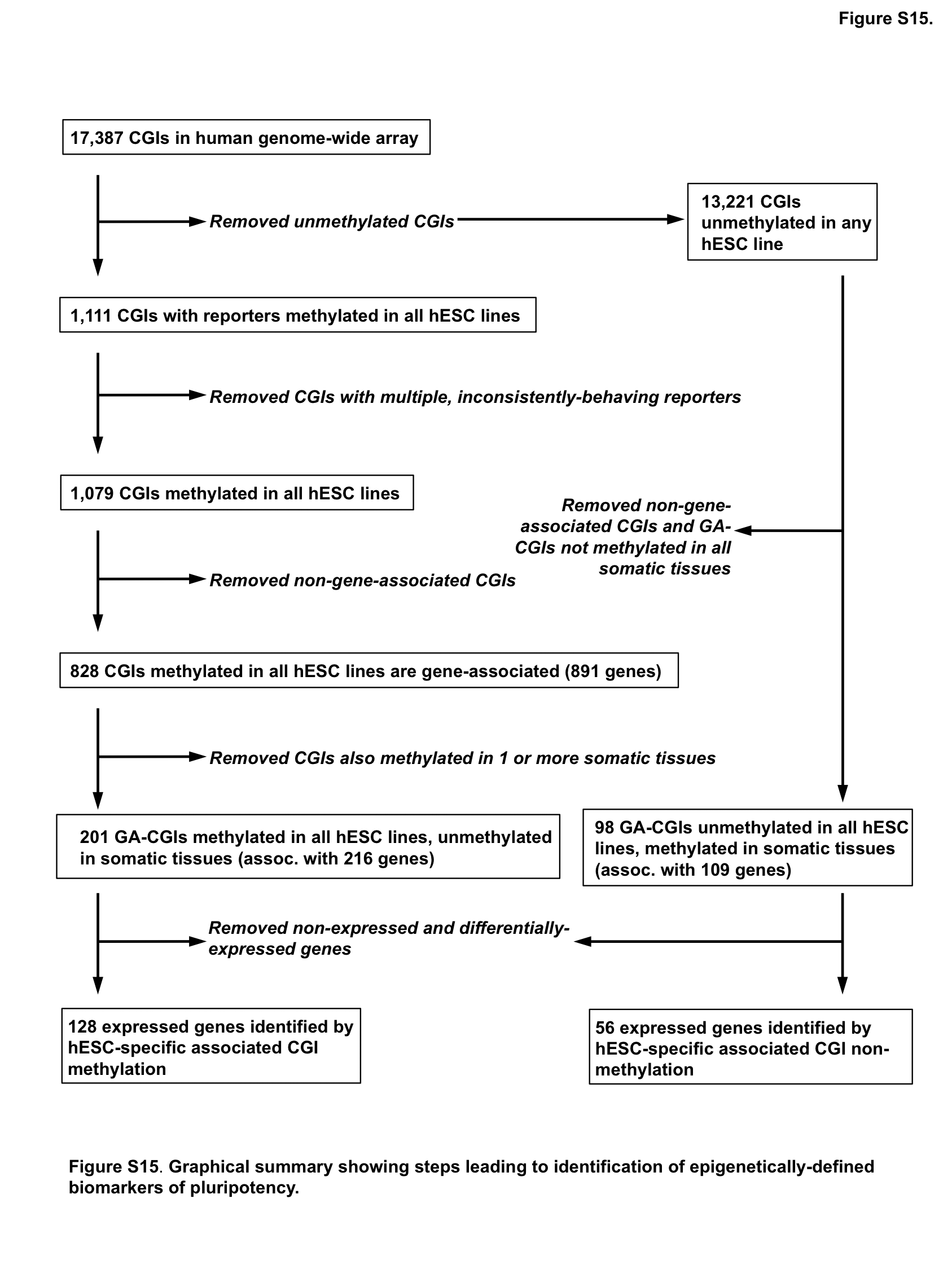

Supplement: S15 Fig — (TIF) [file pone.0131102.s017.tif]
